# Mobile applications for elderly healthcare: A systematic mapping

**DOI:** 10.1371/journal.pone.0236091

**Published:** 2020-07-30

**Authors:** Joseane O. V. Paiva, Rossana M. C. Andrade, Pedro Almir M. de Oliveira, Paulo Duarte, Ismayle S. Santos, Aline L. de P. Evangelista, Rebecca L. Theophilo, Luiz Odorico M. de Andrade, Ivana Cristina de H. C. Barreto

**Affiliations:** 1 Group of Computer Networks, Software Engineering and Systems (GREat), Federal University of Ceará (UFC), Fortaleza, Brazil; 2 Oswaldo Cruz Foundation (FIOCRUZ—CEARÁ), Eusébio, Brazil; Vietnam National University, VIET NAM

## Abstract

**Context:**

The increase in the population aging has brought more significant concern about how proper care will be provided to the elderly in the future. Thus, the development of technological solutions for the health domain has gained more prominence. Joining this scenario to the growing use of mobile devices for daily activities, several mobile applications focused on the elderly healthcare have been developed with healthcare and software engineer professionals involved. However, there is no survey to help both professionals to take decisions on the target of application, elderly profile, empirical validation techniques, among others. Thus, the following question arises: how have mobile applications for elderly healthcare been addressed in the literature in the past years?

**Objective:**

To identify the state of the art in the literature concerned with the development of mobile applications for elderly healthcare, considering healthcare and software Engineering viewpoints.

**Method:**

We performed a systematic mapping conducted by health and software engineering researchers to provide an interdisciplinary investigation of the papers that address mobile applications for elderly healthcare, summarizing the data collected under the following classification: target of application, older adult profile, spatial-temporal distribution, techniques for empirical validation and type of software engineering research.

**Results:**

We found a total of 2533 papers and, after applying our eligibility criteria, we got 149. We observed aspects related to the digital health initiative type, using the classification proposed by the World Health Organization (WHO), the elderly profile prioritized by the application, the spatial-temporal distribution of the studies, the empirical validation type, and the research contribution of each analyzed paper to the software engineering area.

**Conclusions:**

Regarding the WHO classification, we noticed that two categories were more frequently found, Clients and Data Services, and that none of the mobile apps were classified in the Health System Manager category. The data extraction result also reveals that most of the applications found in the literature focused on the independent elderly. Moreover, we observed that most of the studies were proposals of solutions for elderly health and the validation process of these solutions generally consisted of controlled experiments and usability evaluations. At last, the research focused on mobile applications for elderly healthcare has been performed mostly by developed countries.

## Introduction

The increase in life expectancy and the decrease in the birth rate are factors that contributed to the aging of the population [1]. The World Health Organization survey indicates that, by 2050, the number of older adults will be higher than the number of children on the planet [2].

This condition directly affects the number of people available to properly care for the elderly, whether in the informal context (family, friends) or formal context (caregivers, health professionals). This scenario encouraged the development of technologies aimed at increasing the autonomy of the elderly, using tools for self-care, and to support families, friends and health professionals in the area of elderly health [3].

In parallel to this scenario, we have seen the growth in the adoption of mobile applications to perform everyday tasks [4] that previously required the use of personal computers or the user’s displacement. For instance, with a mobile application the grocery shopping can be made in a few clicks using a smartphone, avoiding the trip to the supermarket.

In addition to everyday activities, the use of mobile apps has impacted the way the population has managed healthcare [5]. There are applications to support medication administration, to monitor blood pressure, to schedule medical appointments, among others. This area has been known as mobile health or mHealth and it represents a medical and public health practice supported by mobile devices [6].

Despite the practicality of using mobile applications for the population in general, when it comes to elderly users, some factors need to be considered, such as accessibility. Wildenbos et al. [7] conducted a study to identify the barriers influencing the adoption of mHealth apps for the older adults, their results found four categories of barriers: cognition, motivation, physical ability and perception. Each category present a lot of possible causes that can justify non-adoption of mHealth applications for the elderly, between the reasons identified the most cited was longer learning time and poor visual acuity.

For all categories, it is possible to associate several conditions that an older adult may be exposed to and comprehend which usability factors [8] may be impaired by this condition. Within the motivational barriers, for example, there is a lack of confidence in oneself to use a mHealth app. Older adults may feel insecure when they start using an application and give up using it for fear of damaging something.

The specificities inherent to mHealth applications for the elderly, emphasize the need to know well the profile of older adult to be achieved with the product to be developed. Thus, we must think about the forms to involve this profile in the designing of these applications [9], not only to deal with graphic elements (*i.e*., font size, button colors and position of the elements) [10] but also to project the interaction flow [11] in order to optimize the cognitive process of the target audience.

Many secondary studies have been conducted to better understand what must to be consider to build mHealht applications to the elderly, such as studies on accessibility in mobile applications [11], adoption of mHealth for the elderly [12, 13], mHealth technologies for chronic diseases [14] and interventions of electronic health (*i.e*. e-health) for active aging [15].

However, the literature lacks a study that collects and summarizes the initiatives related to mobile applications for elderly healthcare. This work is performed by both software engineering and health researchers, inspired by Kitchenham guidelines [16], to present a systematic map that characterizes *“how the scientific community has studied the applications of mHealth for the elderly”*. We highlight as main contributions of this work the following observations:

- Most of the papers were in the Personal Health Tracking category, which reveals a high interest in health monitoring solutions and the absence of work on public health systems management;
- We observed that the Independent older adult is prioritized over the Dependent profile. Papers distribution over the years suggests an upward trend from 2014 to 2018 with a high concentration of research in developed countries;
- We found that most of work can be classified as Proposal of solution and they use experiments as a strategy for empirical validation.

As a secondary contribution of this work, the results of this research can illustrate the most common decisions in the development of mHealth apps for the elderly. In this way, the results of this paper provide a comprehensive overview of this area.

The remainder of this paper is organized as follows: in the next section, we present the related works, then we present our methodology and we expose and discuss the results; Next, we describe the threats to validity and how we mitigate them; Finally, we present the conclusions, as well as the future works.

## Related work

As mentioned in the Introduction, we did not find any other systematic mapping that addresses mHealth applications for the elderly. Indeed, there are similar works, but they do not address the research questions raised in this study. Thus, in this section, we briefly present some of these works, highlighting their differences to our proposal.

In [11], the authors performed a literature review on accessibility in mobile applications for the elderly. This review identified methods, approaches, and frameworks for the development and evaluation of mobile applications accessible to the elderly.

Moreover, the authors identified accessibility guidelines for mobile applications and listed the usability barriers faced by the elderly in mobile applications. The work does not focus on mHealth applications for the elderly. Still, its results can support research on this topic and are highly valuable to developers concerned with designing more accessible applications for the elderly on the health domain.

Changizi et al. [17] conducted a literature review considering the range time between 2012 and 2016 to investigate the effectiveness of using mHealth applications to improve the health behaviors of the elderly. The authors searched preferentially for papers that had performed clinical trials and experiments. Then, they analyzed the results of these strategies to verify the effectiveness of the mobile applications. Finally, the results showed that mobile applications are effective tools for improving the health behaviors of the elderly.

In 2012, [18] conducted a literature review to provide an overview of mHealth applications for the elderly. The authors grouped the mobile applications according to the health-related purpose they were intended, such as activities of daily living, falls, risk of falls, among others. The authors provided a summary of data by type of intervention and an overview of mHealth applications for seniors until 2012. They emphasize that mobile applications for elderly healthcare were still in their infancy by the time the research was conducted.

Our work aims to provide a more recent picture of the field, describing how the scientific community has addressed mobile applications focused on the elderly healthcare. In the next section, we describe our methodology and our search strategy to achieve this purpose.

## Materials and methods

A Systematic Literature Mapping (SLM), wich is organized into three macro phases: i) Planning, to build the mapping protocol; ii) Conducting, for data collection, selection and, extraction; and iii) Reporting, for results analysis and discussion. Our methodology was mainly inspired on Kitchenham guidelines and it possible to access our review protocol at http://bit.ly/mHealthAppsElderly.

### Research questions

We applied the PICo strategy (Population, Interest, and Context) [19, 20] to define our Research Questions. We described Population as “scientific papers”; Interest as “mHealth”; and, Context as “older care”.

Thus, we defined the following Primary Research Question (PRQ): **What is the state of art of mobile health applications for elderly healthcare?** This question is detailed by the following Secondary Research Questions (SRQ):

SRQ1 How are the papers distributed in relation to the categories of digital health initiatives made available by WHO in 2018 [21]?
SRQ2 Which is the papers distribution considering the elderly usage profile (*i.e*. independent, when the elderly do not need support to use the application, or otherwise, dependent)?
SRQ3 In the last five years, what is the spatiotemporal distribution of the papers that address mobile applications focusing on elderly healthcare?
SRQ4 What are the empirical validation techniques [22] used to evaluate these papers?
SRQ5 What is the papers distribution focusing on mobile applications for elderly healthcare in relation to the types of research in software engineering [23]?

### Search strategy

We chose four general indexing services based on the representativeness that they have regarding scientific research involving health and technology: Scopus, Web of Science (WoS), Compendex, and PubMed. Together, these indexing services provide access to the most relevant digital libraries for this research, like IEEE, ACM, Elsevier, Wiley, Springer, and MEDLINE digital library [24]. Moreover, we built the search string considering the steps described as follows.

**Step 1**: Identify the keywords in RQ. Considering the PICo strategy, the Population was defined as scientific papers. The keywords for the Interest and Context was defined as mHealth and older care, respectively.

**Step 2**: Select synonyms that could be replaced by the keywords defined in Step 1 and connect them using the OR logical operator. Since the context keyword is composed of two words (“older” and “care”), it was necessary to identify synonyms for both. Also, it important to note that we decided to include “mobile” and “app” terms as synonyms to mHealth in order to cover a higher number of works that would later be refined in the selection stage.

- **mHealth**: *(m-Health OR mhealth OR “mobile health” OR mobile OR app)* AND
- **elderly**: (elder* OR senior OR (older AND (people OR person OR user OR adult OR individual)) AND
- **care**: (health* OR wellbeing OR “wellness” OR “quality of life”).

Table 1 details the process of applying the search string in each database. All searches were performed on April 23, 2019. This information is essential to guarantee the reproducibility of this study.

**Table 1:** Database search string.

Database	Search String	Papers
PUBMED	((((elder*[Title/Abstract] OR senior[Title/Abstract] OR “older people”[Title/Abstract] OR “older person”[Title/Abstract] OR “older user”[Title/Abstract] OR “older adult”[Title/Abstract] OR “older individual”[Title/Abstract])) AND (health*[Title/Abstract] OR wellbeing[Title/Abstract] OR “quality of life”[Title/Abstract] OR “wellness”[Title/Abstract])) AND (“m-Health”[Title/Abstract] OR “mhealth”[Title/Abstract] OR “mobile health”[Title/Abstract] OR mobile[Title/Abstract] OR “app”[Title/Abstract])) NOT (“systematic review”[Title/Abstract] OR “systematic mapping”[Title/Abstract] OR “clinical study”[Title/Abstract]) AND ((free full text[sb] AND full text[sb]) AND (“2014/01/01”[PDat]: “2018/12/31”[PDat]) AND English[lang])	144
SCOPUS	TITLE-ABS-KEY ((elder* OR older people OR older person OR older user OR older users OR older adult OR older adults OR older individual OR older individuals OR senior) AND (health* OR wellbeing OR “quality of life” OR “wellness”) AND (m-Health OR mhealth OR mobile health OR mobile OR app) AND NOT (“systematic review” OR “systematic mapping” OR “clinical study”)) AND (LIMIT-TO (DOCTYPE, “ar”) OR LIMIT-TO (DOCTYPE, “cp”)) AND (LIMIT-TO (PUBYEAR, 2018) OR LIMIT-TO (PUBYEAR, 2017) OR LIMIT-TO (PUBYEAR, 2016) OR LIMIT-TO (PUBYEAR, 2015) OR LIMIT-TO (PUBYEAR, 2014)) AND (LIMIT-TO (LANGUAGE, “English”))	1404
WoS	((((((TS = (elder* OR senior OR “older people” OR “older person” OR “older user” OR “older adult” OR “older individual”) AND TS = (health* OR wellbeing OR “quality of life” OR “wellness”) AND TS = (“m-Health” OR “mhealth” OR “mobile health” OR mobile OR “app”) NOT TS = (“systematic review” OR “systematic mapping” OR “clinical study”))))))) AND LANGUAGE: (English) AND DOCUMENT TYPES: (Article)	388
COMPENDEX	(((elder* OR senior OR “older people” OR “older person” OR “older user” OR “older adult” OR “older individual”) WN KY) AND ((“m-Health” OR “mhealth” OR “mobile health” OR mobile OR “app”) WN KY) AND ((health* OR wellbeing OR “quality of life” OR “wellness”) WN KY) NOT ((“systematic review” OR “systematic mapping” OR “clinical study”) WN KY))	597
	**Total**	2533
	**Total without duplicates**	1812

### Eligibility criteria and study selection

We defined the following eligibility criteria: (i) Must present a primary study covering a mobile application focused on elderly healthcare; (ii) Be written in English; (iii) Be full available at the Internet; (iv) Papers with five or more pages; (v) Be published in a workshop, conference, journal, magazine or newspaper between 2014 and 2018. To provide a recent overview of studies on mHealth applications for the elderly and to get focused results, we have specified the period from 2014 to 2018 to cover the last five years. [25].

**Fig 1:**
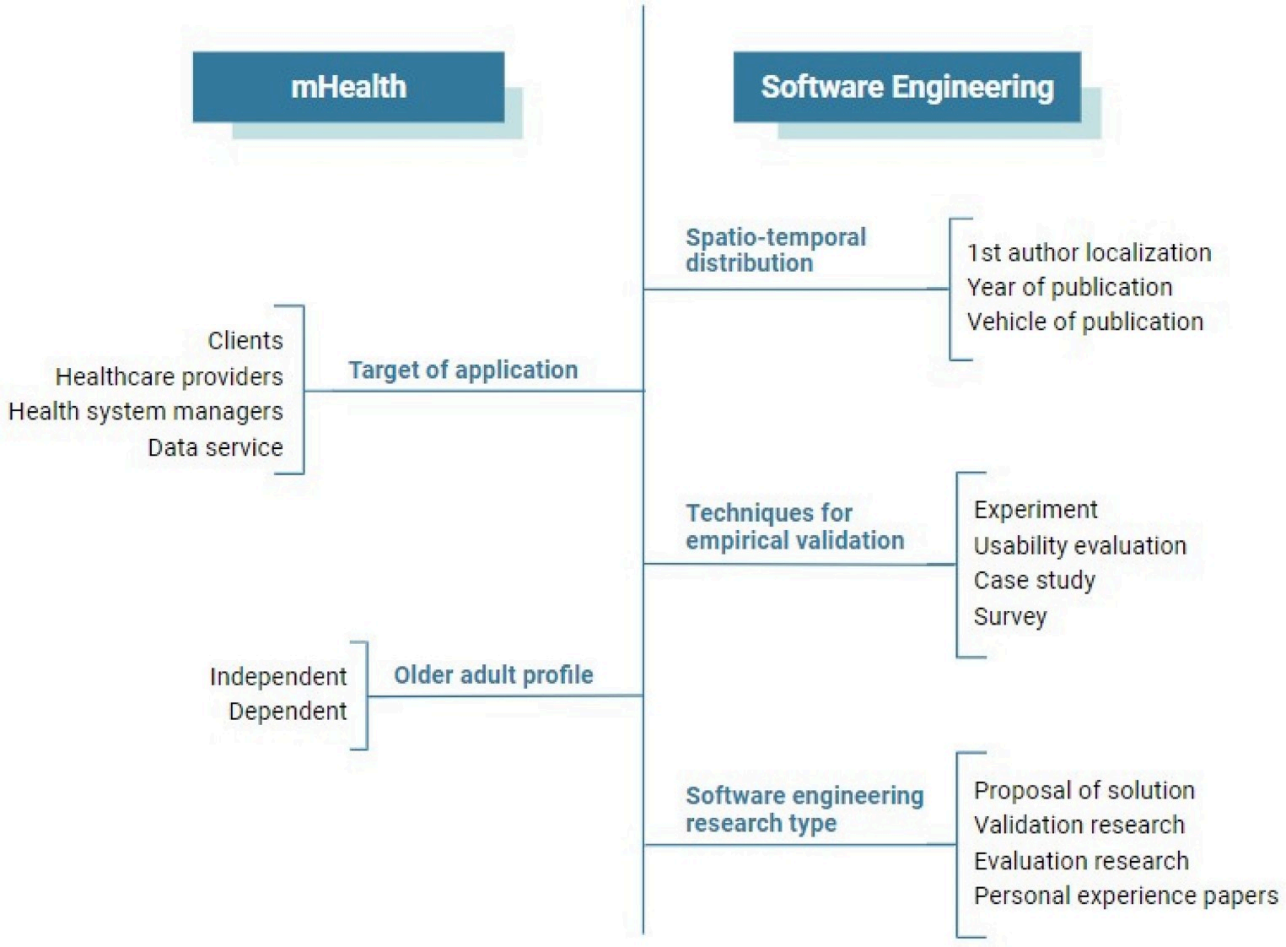
Classification scheme for the data summarization.

We performed five steps during the study selection:

- **Step 1**: we applied the search strategies in all selected databases to collect the primary studies. The strings are listed in Table 1.
- **Step 2**: all researchers involved in the selection stage read titles and abstracts from 10% of the primary studies applying the eligibility criteria for study selection.
- **Step 3**: the results obtained by each researcher were compared using the Kappa statistical test (agreement analysis) [26]. The agreement level was considered very good, with a Kappa value of 0.8912. Even so, we held a meeting to discuss the divergent points.
- **Step 4**: after selecting the papers, we performed the full reading process and data extraction. Each paper was read by at least two researchers: a technology expert and a health expert. Thus, data extraction occurred considering two perspectives: health and technology. Also, the full reading allowed a review of the selection step. We conduct weekly meetings to discuss extracted data and unified the answers.
- **Step 5**: After a final round of review, to ensure the consistency of the data obtained, we created a data summarization under the following classification: target of application, older adult profile, spatiotemporal distribution, techniques for empirical validation, and software engineering research type. We illustrated this classification scheme in Fig 1.

## Results

**Fig 2:**
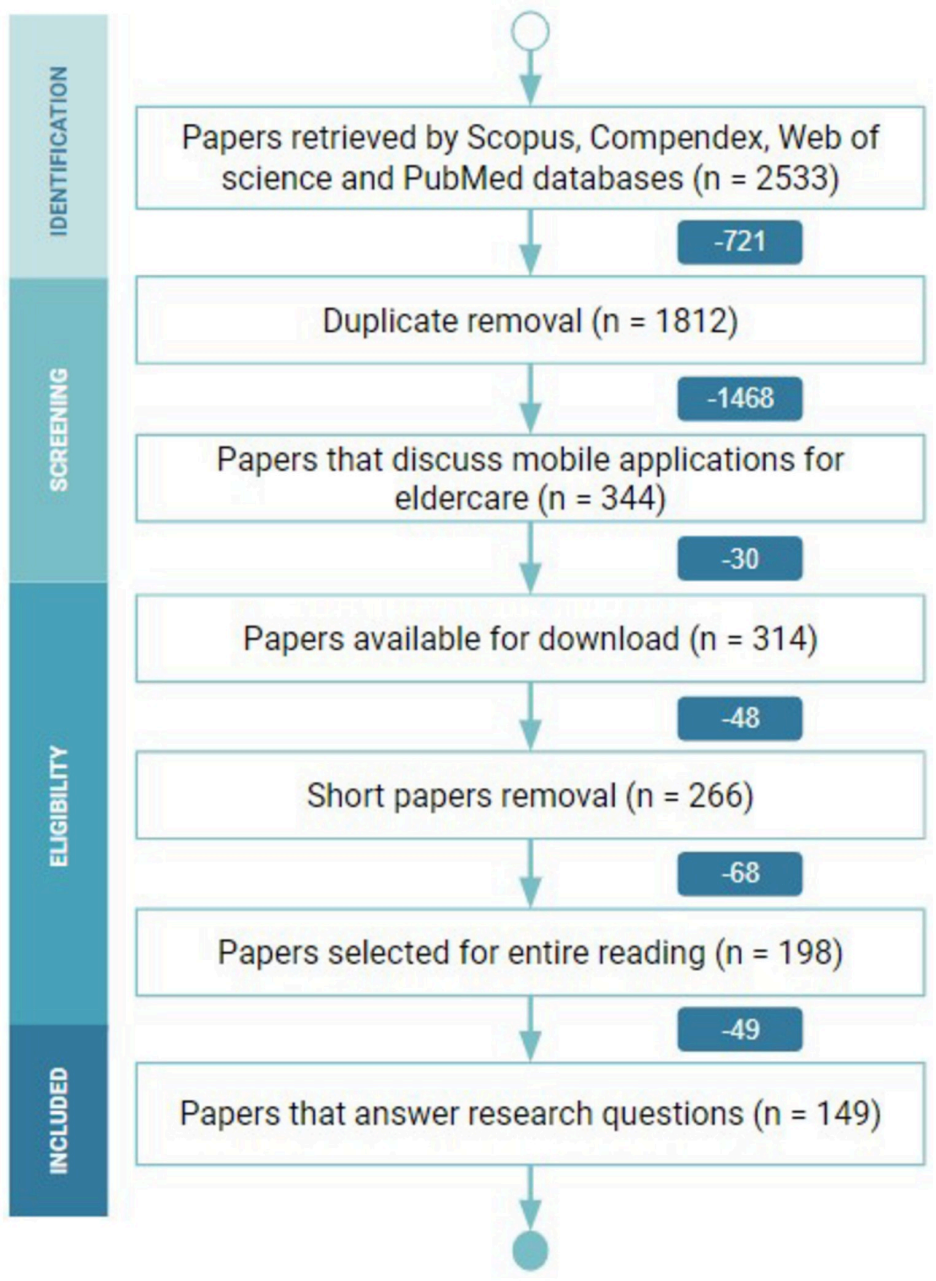
PRISMA flow diagram of the different phases of the systematic mapping.

Initially, we collected a total of 2533 papers. With duplicates removal, we got 1812 papers. After applying the eligibility criteria, we selected 198 for the entire reading. After that, we removed 49 papers that did not answer our research questions. Thus, we end up with 149 papers. Fig 2 illustrates this process using the PRISMA Flow [27], a diagram created to map the number of papers during the phases in a literature review (*i.e*. Identification, Screening, Eligibility, and Included).

The detailed results of this mapping (*e.g*. the complete list of the papers, the raw extraction process data, and other graphs) are available at http://bit.ly/mHealthAppsMapping.

### SRQ1: DHI classification

To better understand the state of the art of mobile applications focused on elderly integral healthcare, we decided to analyze five aspects. The first aspect refers to the WHO Digital Health Initiatives (DHIs) classification. It represents a WHO initiative to classify the use of mobile technologies to support health systems [21]. DHIs have four macro classes:

1. Interventions for **Clients**, which includes both systems focused on healthcare users and caregivers of clients who use health services.
2. Interventions for **Healthcare Providers** with solutions focused on who delivers health services.
3. Interventions for **Health System Managers** with solutions that involve public health systems management, such as GISSA [28–30], a Brazilian Web system that captures data from the Unified Health System and is based on business intelligence concepts, being able to generate alerts and help in decision-making.
4. Interventions for **Data Services**, which consists of interventions related to data collection, management, use, and exchange.

Each of DHIs classes has categories to fine-tune the mHealth technology classification [21].

**Fig 3:**
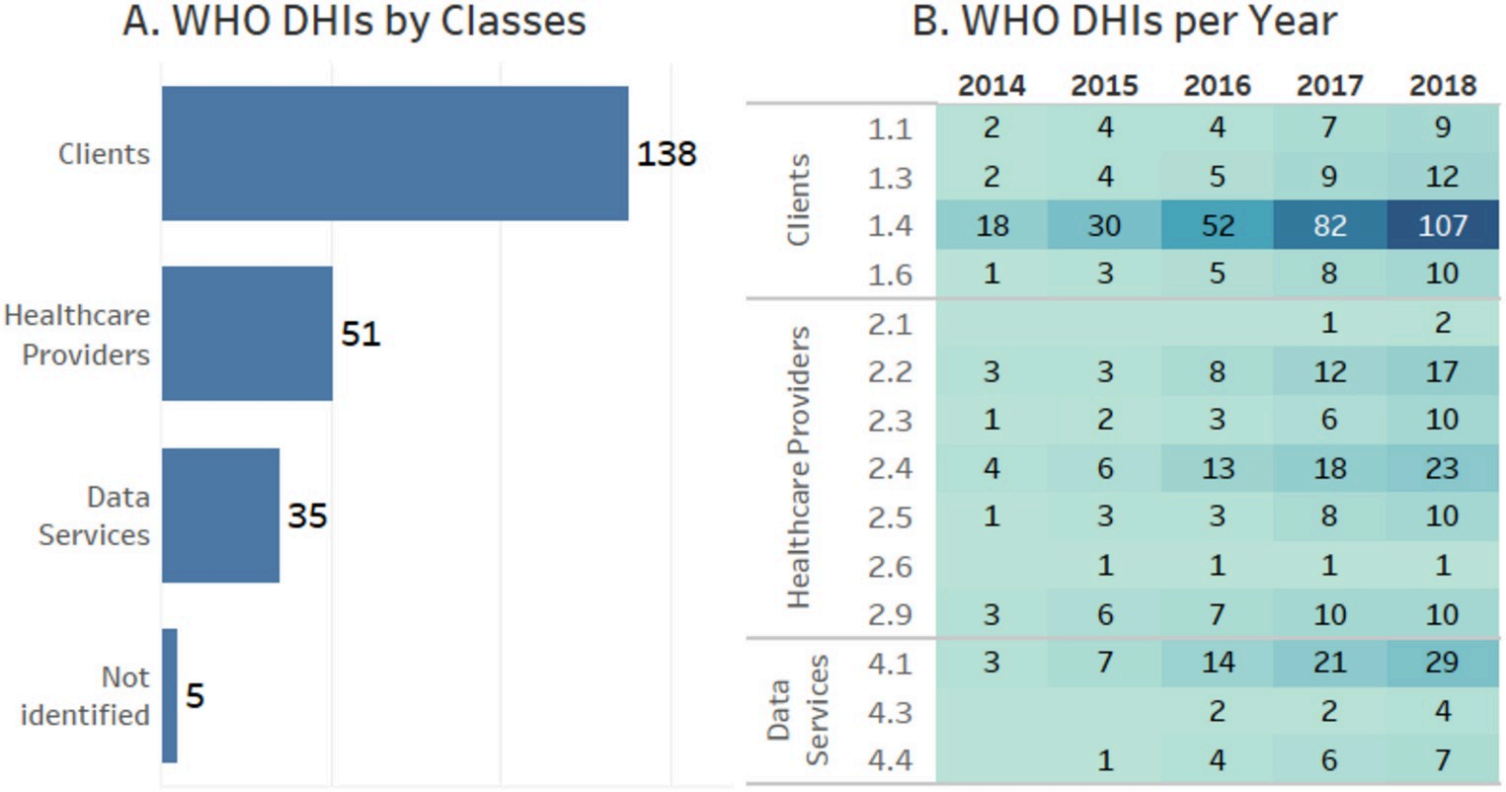
In this Figure we have: A. a visualization of paper distribution by DHI classes; and, B. a heatmap with cumulative total of paper over the years by DHI classes and categories.

As presented in (Fig 3A), the Interventions for Clients has the most papers (138 of 149). The category of this class with the most papers is 1.4 Personal Health Tracking that includes applications in which customers use smartphone sensors, health records, and wearables to monitor their health. The second class with most papers is Interventions for Healthcare Providers (51 papers). For this class, the highlighted category is 2.4 Telemedicine that propose the delivery of healthcare services at distance. The Interventions for Data Services class has 35 papers, and 29 of these papers are in the 4.1 Data Collection, Management, and Use category. We did not find any paper reporting mobile health Interventions for Health System, and it was not possible to identify any class or category for 5 papers.

Note that one paper may belong to more than one class or category. For example, the paper proposed by [31] was classified as Interventions for Clients, because it presents an app for elderly care and follow-up, and as Interventions for Data Services, because it deals with data collection of sensors.

Fig 3B presents a heatmap with the cumulative total of papers in DHIs classes and categories. We omitted some classes and categories (*e.g*., Interventions for Health System or Resource Managers), because no paper found in this study was classified in these classes and categories. To improve the visualization, we omitted them from the graphics.

### SRQ2: Elderly usage profile

**Fig 4:**
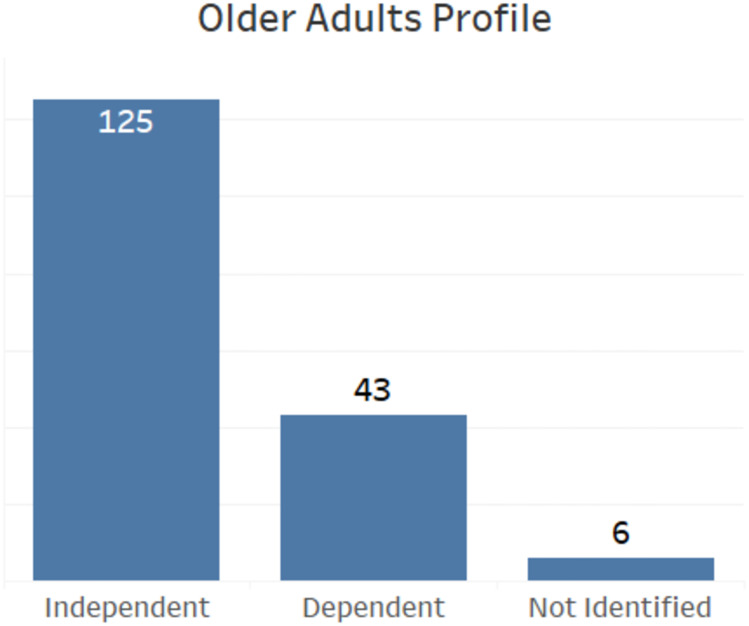
Bar chart with the number of papers by older adults profile.

SRQ2 guided the investigation to understand the papers distribution considering the older adult usage profile. We used an adaptation of the dependency concept proposed by [32] to classify papers on Independent, Dependent, or Not Identified profiles. We classify as Dependent, solutions proposed for elderly who have limitations that prevent them from using all application functions, *i.e*., the elderly would need support to use the app. In this case, the benefit is indirect. We classified as Independent when the elderly can use the solution without any help. In Fig 4, it possible to observe that most papers were classified with the Independent profile (125 of 149).

Forty-three papers were classified with the Dependent profile and, in 6 papers, it was not possible to identify any profile. Note that the same study can address both profiles (Dependent and Independent).

### SRQ3: Spatiotemporal distribution

**Fig 5:**
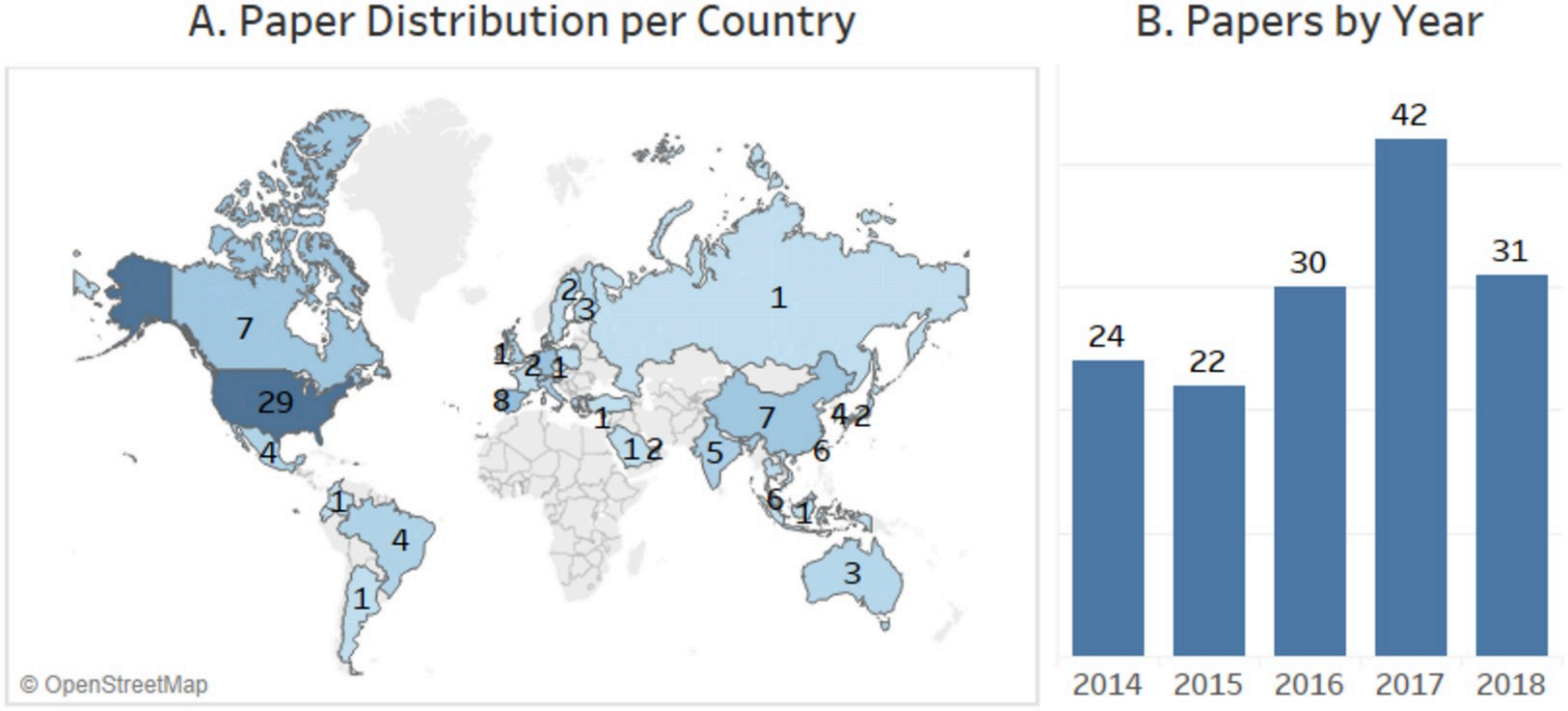
This Figure presents A. a global map showing the total of papers by country; and, B. a graph with the amount of paper per year.

To deeply understand the state of art of mHealth applications focused on older adults, we analyzed the papers spatiotemporal distribution in the last five years. In response, it was observed (Fig 5B) that the papers number presents an upward trend from 2014 to 2018 (an increase of 29.17%).

Global map (Fig 5A) shows that United States has always been ahead on the number of publications in this area (29 papers from 2014 to 2018). After the United States, Spain have 9 papers followed by Portugal and Germany with 8 papers each. Another observation from our data is the increase of publications in Asia between 2015 and 2016.

We also analyzed the conferences and journals of our selected studies. Table 2 shows the most recurrent of them. To improve data visualization, we removed those with just one paper. The ACM International Conference Proceeding Series has nine papers. Two journals with six studies each follow it: Lecture Notes In Computer Science and JMIR MHealth and UHealth, respectively.

**Table 2:** Most recurrent conference and journals considering our selected studies.

Conference/Journal	Type	Papers
ACM International Conference Proceeding Series	Conference	7
Lecture Notes In Computer Science	Journal	6
JMIR Mhealth And Uhealth	Journal	6
IEEE International Conference on Healthcare Informatics, ICHI	Conference	4
IEEE International Symposium On Medical Measurements And Applications, MEMEA	Conference	4
Journal of Medical Internet Research	Journal	3
Studies In Health Technology And Informatics	Journal	3
Human Factors And Ergonomics Society	Journal	3
JMIR Research Protocols	Journal	3
IEEE International Conference On E-health Networking, Applications And Services, HealthCOM	Conference	2
Journal of Ambient Intelligence And Smart Environments	Journal	2
Journal of Medical Systems	Journal	2
Telemedicine and E-health	Journal	2
Sensors	Journal	2
IEEE International Conference On Computer And Information Technology; Ubiquitous Computing And Communications; Dependable, Autonomic And Secure Computing; Pervasive Intelligence And Computing	Conference	2
International Conference On Human-computer Interaction With Mobile Devices And Services, MobileHCI	Conference	2
International Conference On Information And Communication Technologies For Ageing Well And E-health	Conference	2
International Bcs Human Computer Interaction Conference, HCI	Conference	2
Communications In Computer And Information Science	Book Serie	2
Advances In Intelligent Systems And Computing	Book Serie	2
Amia, Annual Symposium Proceedings. Amia Symposium	Conference	2
International Journal of Medical Informatics	Journal	1

### SRQ4: Empirical validation techniques

**Fig 6:**
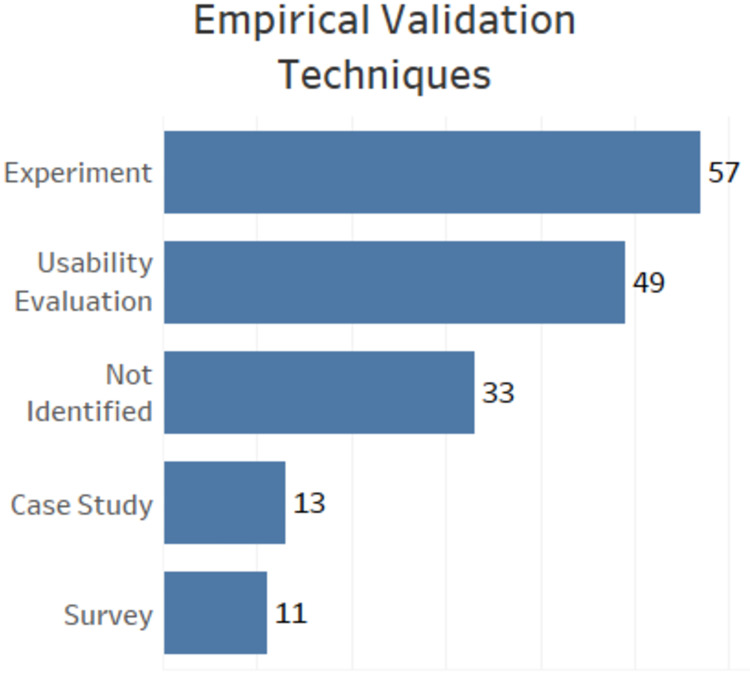
Bar chart with data regarding empirical validation techniques.

SRQ4 focuses on the empirical validation techniques used by the researchers. For this question, we used the classification proposed by [22] including Usability Evaluation. We decided to include it because many papers validate their proposals using usability evaluations. Fig 6 shows that most papers use experiments as an empirical validation method.

At the end, we have 57 papers classified like Experiments, 49 papers like Usability Evaluation, 13 papers like Case Study and 11 papers as Surveys. In 33 papers, we did not find the empirical evaluation method clearly described. Thus, they were classified as Not Identified for this criterion.

### SRQ5: Types of research in software engineering

Regarding the types of software engineering research (proposed by [23]), Proposal of Solution represents papers that propose solutions arguing about its relevance, but without presenting a robust evaluation. Validation research papers seek to investigate aspects of a solution through experiments, simulations, prototyping, proofs, or mathematical analysis. Evaluation research, in turn, investigates a problem in practice usually through case studies, field experiments, or surveys. Finally, Personal Experiences Papers emphasize the authors’ personal experience on a particular topic.

**Fig 7:**
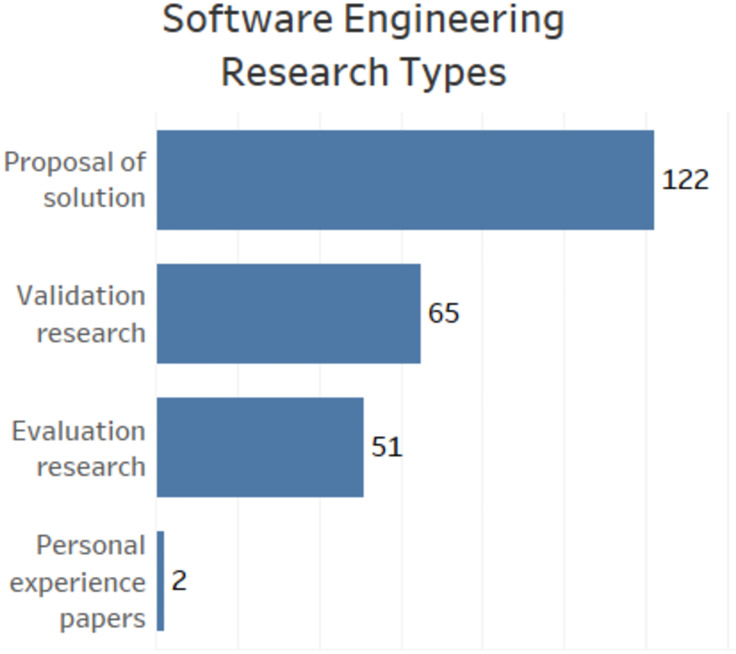
Graphic presenting data related to the type of software engineering research.

Fig 7 shows that we found 122 papers classified as Proposal of Solution; 65 papers of Validation research type; 51 papers classified as Evaluation research, and two papers reporting Personal experiences. It is important to note that the same paper can be classified with more than one type of software engineering research.

## Discussion

This review was conducted to answer the following Primary Research Question: how the scientific community has studied the applications of mHealth for the elderly? However, to better understand the aspects involved in this question, we decided to split this macro question into five Secondary Research Questions (SRQs). The answers came from the detailed analysis of 149 from 2533 papers that returned from the initial search.

Regarding SRQ1, this secondary question helped us to understand the paper distribution from the WHO perspective for Digital Health Interventions. The WHO DHI classification, although recent (published in 2018), covers a wide range of mobile technologies aspects designed to support the healthcare systems needs. We found some expected behaviors, *e.g*., a greater interest in researches focused on healthcare clients and an outlier in the number of papers proposing solutions for Personal Health Tracking. In fact, the development of mobile applications is still very focused on providing services to the end customer of health services. Also, advances in the development of new sensors combined with the cost reduction of these devices have facilitated access to solutions for monitoring vital signs and other health measures. We believe that this category of applications will be strong for an extended period.

Another finding of SRQ1 was the absence of papers addressing mobile applications focused on health care for the elderly in the Health System Managers class. One possible reason for that, is the difficult of available data for research in such kind of systems, since they are usually commercial systems and managed by the government or private companies. So, there is a research gap of studies dealing if this kind of systems. We argue that the development of open-source platforms for the management of health systems can, for example, support studies on new methods and approaches focused on optimizing processes in this area.

We also highlight that the Healthcare Provider Interventions class is promising for future research due to the growing interest in solutions that deliver healthcare at a distance and that can support the coordination of emergency response to local or global health issues. The categories in which we observed a growing trend were telemedicine (to provide healthcare services at distance), client health records (to manage client health information), decision support (to assist healthcare providers in making diagnosis and treatment decisions), and medication management (to facilitate the management of prescriptions, including tracking prescription orders and monitoring physical consumption of medication). We believe such categories may get even more attention as consequence of Internet of Medical Things [33], which have changed how health data is collected and stored.

Regarding to SRQ2, we evaluated the papers trying identify if the paper was developed considering the independent elderly profile, which does not need someone else’s support to use the application, or considering the dependent elderly profile, *i.e*., the one who needs the caregiver support to use all the functions of the application. The answer was that the independent elderly profile is prioritized in the mobile health applications area. The number of papers dealing with the dependent elderly profile is low. Thus, another research gap is related to the quality of life of older adults who depend on caregivers.

Analyzing the SRQ3, we observed that the number of papers published between 2014 and 2018 presented an average of 29.8 and standard deviation of 7.82. These metrics show that this area has been kept warm and with a slight growing trend. In the spatial perspective, we found a higher number of publications in developed countries (*e.g*., United States and Germany).

Another fact that deserves attention is the absence of papers published by African researchers. A hypothesis for this latter is that mHealth is a relatively new area in Africa [34]. Thus, the number of studies conducted by researchers from this region on mHealth for elderly may start to grow considering the improvements of the Human Development Indexes (HDI), and the seek for aging with greater well-being.

Regarding the conferences and journals in which the studies were published, we observed that there was no predominance. However, the total number of papers published in journals (81) is higher than the papers published in conferences (64). Moreover, only four studies were published in Book Series. In Table 2, it is possible to observe the most recurrent conferences. They probably represent suitable scientific events for discussing new proposals in this area.

SRQ4 sought to find out which empirical validation techniques are commonly used in the papers included in our mapping. In response, we found that most papers use controlled experiments for empirical validation. This strategy is marked by high rigor in the execution control and measurement. This strategy may be widely used due to ethical issues involving researches with the elderly in uncontrolled environments. For example, it is almost impractical to evaluate a fall detection mobile application with older adults in an uncontrolled environment or when the researchers cannot guarantee the participants physical integrity.

The second most used technique is usability evaluation. This strategy is not part of the paper proposed by [22] because, depending on the instrument used (*e.g*., observation, interviews, questionnaires, laboratory experiments, focus group), a usability evaluation may have characteristics of a controlled experiment, survey or case study [35]. However, we decided to include this strategy as a distinct item in the extraction form because of the importance that usability has in developing mobile applications for seniors [7]. In general, there are still many older adults who believe that these technologies are hard to use [36]. So, conducting a usability assessment becomes essential for the proposed mHealth technology to have good adherence by the elderly.

Another finding in the SRQ4 was the high number of papers in which it was not possible to identify the empirical validation technique. This can indicate that there is room for proposals of new approaches that help researchers and practitioners to use empirical evaluation methods. We also highlight that due to the high cost of these empirical validations, the number of studies about formal verification and validation techniques, and runtime testing for mHealth should increase.

For SRQ5, we ranked the papers according to the software engineering research types proposed by [23].

We identified that the class with the most papers is Proposal of solution, followed by Validation research, and Evaluation research. This rank indicates that much of the paper proposes solutions without presenting a robust assessment. This data is reinforced by the number of papers in which we did not identify any empirical validation technique. Therefore, it is still possible to strengthen this research area proposing solutions with more robust evaluations.

Thus, we have as an answer to our Primary Research Question that the state of art for mobile applications focusing on elderly healthcare is concentrated on developing personal health tracking solutions for the independent elderly profile. These solutions are generally evaluated through controlled experiments and usability evaluations. However, there are many published papers that do not have robust evaluation. The mHealth area has been kept active mainly due to research conducted in developed countries. We did not find papers published by African researchers or papers focusing on public health systems.

**Fig 8:**
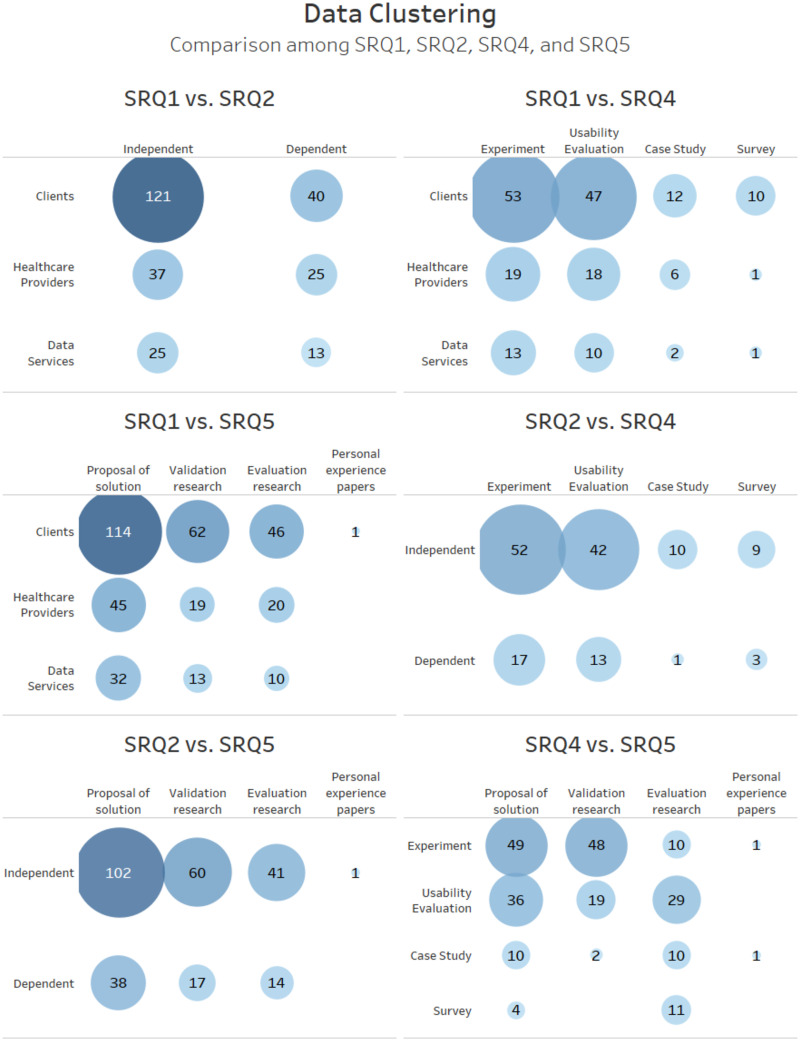
Data clustering presenting correlations among SRQ1, SRQ2, SRQ4, and SRQ5.

Fig 8 shows the correlation between the WHO classification (SRQ1), elderly profiles (SRQ2), empirical validation techniques (SRQ4) and the software engineering research types (SRQ5). We did not include SRQ3 in this correlation, because we sought to analyze the spatiotemporal distribution more broadly. This data clustering helps us to observe trends and gaps in mobile health applications for older adults. For example, there is a high number of applications for independent elderly clients that were validated through experiments or usability evaluations. Few works are classified as personal experience papers, and there is a short number of case studies in studies focused on healthcare providers.

Finally, we highlight that all mobile health applications found in the 149 papers selected in this work are described in a table present in our supporting information section.

### Challenges and future perspectives

According to [37], the mHealth apps market will be valued at almost USD 18 billion in 2025. This forecast points out the strengthening of smartphones usage for healthcare and the emergence of new challenges in this domain. Our previous discussion helped us to draw some challenges and future perspectives for the development of mobile health applications. These challenges can be analyzed considering the perspective of both technology and health professionals. We highlight the following challenges:

- **Quality assurance**: as new technologies arise, the methods for quality assurance of mobile health applications also need to evolve. For example, the reduction in costs related to health tracking devices has facilitated their access to a large number of users. All of these devices together increase the complexity and dynamics of the environment monitored by mHealth applications [38]. This new scenario requires greater versatility, flexibility, and resilience from these applications. Such characteristics can be achieved with self-adaptation mechanisms [39]. However, the use of self-adaptation presupposes new methods and approaches for quality assurance [40]. We highlight runtime testing [41] that is defined by the execution of tests in the final system environment. There are also studies about formal proof, which is based on mathematical calculations, and model checking, which seeks to evaluate properties of a system in all reachable states, to provide evidence of the quality of these systems. This topic represents an interesting challenge for future research.
- **Data privacy and security**: privacy and security of data from patients, physicians, and other health professionals are essential due to its high sensitivity. Also, due to the large amount of data created by healthcare devices, there is a need to process this information with big data techniques. However, combining privacy and security with efficient data processing is not easy. This challenge is also relevant from the users’ perspective who do not wish to use systems that can expose their information, and considering the health professionals’ perspective who need to ensure that patient data was not violated for their correct analysis during health treatments. Thus, researchers have proposed different approaches to this challenge, such as data anonymization [42] and data reliability with blockchain [43].
- **Data Mining and Machine Learning**: these areas play a fundamental role in the so-called Healthcare 4.0 (term derived from Industry 4.0 and used to characterize the new health model that is beginning to emerge) [44]. With Data Mining and Machine Learning techniques, it is possible to obtain valuable insights and patterns about patient data for disease prediction [45], emergency detection [46], regulation of health systems [47], among others. In our data, we found, for example, some mobile applications that use machine learning to detect falls in the elderly. In the next years, with the sharp growth in available health data and the need for optimized processes to reduce healthcare costs, this research area will be prominent.
- **User acceptance**: after observing i) the growing number of papers that report the execution of usability evaluations, ii) the presence of studies that sought to evaluate the acceptance of mHealth apps in health treatments, and iii) investigations on development processes that enable an active participation of final users, we highlight as a challenge the investigation of processes and strategies to increase the users’ acceptance regarding the mHealth apps usage in their treatments [48]. This challenge is even more complex considering older adult users. Health researchers have reported that many health programs depend on commercial mHealth applications, but that there is a lack of studies focused on validating and discussing the real benefits of these applications [49]. Thus, we believe that user-centered development will be further strengthened in next years.

Finally, based on the results of our systematic mapping, we highlight as questions for future perspectives:

1. How to improve the processes of quality assurance related to the development of mobile health applications?
2. Is it possible to integrate into the mHealth apps workflow concepts of edge and mist computing to provide data mining with privacy and security for the patients?
3. What strategies can be used to increase the number of Evaluation Research papers, *i.e*., more rigorously evaluations in practice?
4. How can design and usability techniques be used to improve the development of applications for the dependent elderly profile?
5. How can mHealth applications help to improve the quality of life of older adults who depend on caregivers?

## Threats to validity

An essential issue of quality and rigorous research is the discussion of threats to validity [50, 51]. This discussion reinforces the work maturity by exposing the planning undertaken to address the threats that could affect this mapping.

Considering the planning phase, we identified as threat to validity i) the definition of Research Questions; ii) the lack of standard terminology between health and software engineer areas, which impact on the search string choice; iii) the use of automated search mechanism; and iv) the restricted time span versus inadequate number of papers. We mitigate these threats by utilizing the following strategies: a collaborative building of a rigorous protocol with healthcare and software engineer professionals; and performing multiple tests to adjust the search string and check the results consistency. Despite of these decisions, we believe that did not impact on the papers representativeness, because the initial search returned 2533 papers from the most representative digital libraries for health and technology areas.

In the conduction phase, the main threat is directly related to the researcher’s bias in selecting and extracting from primary studies. We mitigate this issue by establishing in the selection phase: an agreement analysis among the reviewers using a random set of 10% of all papers; and periodic meetings for perspective alignment. At the extraction phase, the process included:

1. initial training for all reviewers about each extraction form field;
2. double analysis of all papers (one software engineer professional and one health professional);
3. weekly meetings to discuss differences in the extraction of each paper.

Other threats were also considered, for example, duplicate or inaccessible papers. To mitigate these issues, we excluded unavailable papers or short papers, and we used a tool (TheEnd—Systematic Mapping Tool: https://easii.ufpi.br/theend) that helped us remove duplicates. Finally, in the reporting phase, for strength the conclusions, our results were evaluated by health and technology experts.

## Conclusion

As the world population ages, increases the interest in technological solutions that help us achieve healthy and active aging. In this regard, several mobile technologies have been proposed over the last five years.

This work aimed to investigate how the scientific community has studied mobile applications focused on the elderly healthcare. For this purpose, we conducted a systematic mapping following the guidelines proposed by [24], which is widely used in the conduction of literature reviews in the field of Software Engineering.

By applying our search strategy in 4 databases (Web of Science, Scopus, Compendex, and PubMed), we found 2533 studies. We submitted this set of studies to a selection process, and based on a set of eligibility criteria, we obtained a set of 149 papers addressing mobile applications focused on the elderly healthcare.

We analyzed the selected studies in order to understand the following points: (1) the type of initiative according to the WHO Classification of digital health interventions, (2) the profile of the elderly contemplated (3) spatiotemporal behavior of studies on mobile applications focused on elderly health in the last five years, (4) type of contribution to Software Engineering provided by these studies, and (5) empirical validation strategies most frequently used in works addressing mobile applications for the elderly healthcare.

We concluded that most mobile applications focused on the health of the elderly belong to the Personal Health Tracking category, which reveals a great interest in health monitoring solutions from mobile applications. Analyzing the description of the applications to understand if the application focus on the use by the older adult, or use by other people involved in health care of the elderly, we observed that most of the mobile applications found in the literature focused on the elderly as end-user.

Regarding the space-time behavior, we identified that there was significant growth in the interest for mobile applications for the elderly healthcare in the scientific community between the years 2014 and 2018 and we noticed a high concentration of works being conducted by the United States, and by European countries, such as Portugal, Spain and Germany. Furthermore, we concluded that most of the studies analyzed contribute to Software Engineering field with proposals for new solutions, and that the validation strategy most adopted by these studies was the execution of experiments.

We believe that the summarization of the results obtained in this study can benefit both industry and the scientific community. This systematic mapping provides an overview of how the area of mobile applications focused on elderly health has been organized. Thus, we bring contributions to the digital health community by presenting trends and gaps regarding the WHO DHIs classification and the older adult profile. Similarly, we also bring contributions to software engineering researchers working in this area. In this case, it was possible to observe the types of software engineering studies and the methods used for empirical validation. Together, these contributions can support multidisciplinary teams that seek to propose new solutions for this domain.

As future works, we intend to conduct a tertiary study searching for mobile applications focused on the health of the elderly available at Play Store and App Store and also by white papers that address this same theme. Since the profile prioritized by the mobile applications we analyzed was the independent elderly, we also intend to conduct a study on usability assessment methods for mobile applications focused on older adults.

## Supporting information

S1 File Systematic mapping protocol. (DOC)

S2 File PRISMA checklist. (DOCX)

S3 File List of mHealth applications for elderly identified. (PDF)
