# Supplementary material for: Mobile applications for elderly healthcare: A systematic mapping
Source: PLoS One. 2020 Jul 30;15(7):e0236091. doi: 10.1371/journal.pone.0236091 (PMC7392241; doi:10.1371/journal.pone.0236091)
Supplement: S1 File — (DOC) [file pone.0236091.s001.doc]

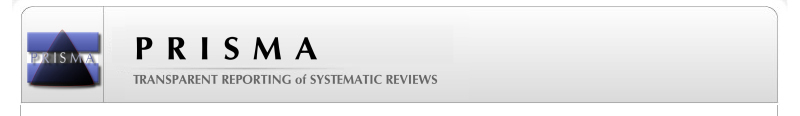
**PRISMA 2009 Flow Diagram**

**Screening**

**Included**

**Eligibility**

**Identification**

Records identified through database searching
(n = 2533 )

Additional records identified through other sources
(n = 0 )

Records after duplicates removed
(n = 1812 )

Records screened
(n = 266 )

Records excluded
(n = 1546 )

Full-text articles assessed for eligibility
(n = 198 )

Full-text articles excluded, with reasons
(n = 49 )

Studies included in qualitative synthesis
(n = 149 )

Studies included in quantitative synthesis (meta-analysis)
(n = 0 )
