# Supplementary material for: Mobile applications for elderly healthcare: A systematic mapping
Source: PLoS One. 2020 Jul 30;15(7):e0236091. doi: 10.1371/journal.pone.0236091 (PMC7392241; doi:10.1371/journal.pone.0236091)
Supplement: S3 File — (PDF) [file pone.0236091.s003.pdf]

Paper ID	Title	Authors	Country	Year	Description
1	Repetitive memorization mobile application development for elderly memory recall	Hamiz, M. and Bakri, M. and Haron, Haryani and Sabri, Sabiroh Md and Jamil, Nursuriati	Malaysia	2014	This mobile application assists the cognitive process of memorization, using Quranic Verses as a basis.
2	Personalizable smartphone-based system adapted to assist dependent people	Igual, Raul and Plaza, Inmaculada and Medrano, Carlos and Rubio, Maria Angeles	Spain	2014	An assisted living mobile application in which caregivers receive instructions from an integrated web service.
3	A mobile application for cognitive screening of dementia	Zorluoglu, Gokhan and Kamasak, Mustafa E. and Tavacioglu, Leyla and Ozanar, Pinar O.	Turkey	2017	A mobile application that applies a battery of multidisciplinary tests to detect dementia.
4	A mobile healthcare solution for ambient assisted living environments	Rodrigues, Daniel F. M. and Horta, Edgar T. and Silva, Bruno M. C. and Guedes, Fabio D. M. and Rodrigues, Joel J. P. C.	Portugal	2016	An AAL application that uses biofeedback monitoring using body sensors for data collection offering support for remote monitoring.
5	A Spanish Pillbox App for Elderly Patients Taking Multiple Medications: Randomized Controlled Trial	Joaquin Mira, Jose and Navarro, Isabel and Botella, Federico and Borras, Fernando and Nuno-Solinis, Roberto and Orozco, Domingo and Iglesias-Alonso, Fuencisla and Perez-Perez, Pastora and Lorenzo, Susana and Toro, Nuria	Spain	2018	A medication self-management app for older adults patients taking multiple medications.
6	A cloud medication safety support system using QR code and Web services for elderly outpatients	Tseng, Ming-Hseng and Wu, Hui-Ching	Taiwan	2014	An Android application thats provides some primary functions including reminders for medication, assistance with pill-dispensing, recording of medications, position of medications and notices of forgotten medications for elderly outpatients.
7	A mobile/web app for long distance caregivers of older adults: functional requirements and design implications from a user centered design process	Williamson, S.S. and Gorman, P.N. and Jimison, H.B.	USA	2015	A mobile/web application focused on remote assistance to caregivers.
8	A smartphone-based fall risk assessment tool: Testing Ankle Flexibility, Gait and Voluntary Stepping	Guimaraes, Vania and Ribeiro, David and Rosado, Luis and Sousa, Ines	Portugal	2014	A mobile application that uses the smartphone's sensors to detect falling tendency in older adults.
9	A wearable smartphone-based system for electrocardiogram acquisition	Depari, A. and Flammini, A. and Sisinni, E. and Vezzoli, A.	Italy	2015	A mobile application that single lead electrocardiogram trace acquisition system.
10	Agreement in gait speed from smartphone and stopwatch for five meter walk in laboratory and clinical environments	Soangra, Rahul and Lockhart, Thurmon E.	USA	2016	A Smartphone app computes gait speed in older adults cardiovascular disease (CVD) patients.
11	Alive Inside: Developing mobile apps for the cognitively impaired	Nezerwa, Martine and Wright, Robert and Howansky, Stefan and Terranova, Jake and Carlsson, Xavier and Robb, John and Coppola, Jean F.	USA	2017	This mobile app stimulates the memory of those suffering of Alzheimer and dementia through music of their youth.
12	An android-based heart monitoring system for the elderly and for patients with heart disease	Pierleoni, Paola and Pernini, Luca and Belli, Alberto and Palma, Lorenzo	Italy	2017	An app that receives information by a heart rate monitor to detect stress states and arrhythmia. This app is suitable for the elderly and by patients with heart disease provide and it is capable to monitor user's health and generate emergency alerts.
13	Application of information technology to develop the system for solitary death	Takami, Shunki and Torii, Ippei and Ishii, Naohiro	Japan	2014	Anshin is an app to prevent solitary deaths, it is integrated to a medical record system that manage the health of the elderly living alone.
14	An integrated and digitized care framework for successful aging	Dasgupta, D. and Feldman, K. and Waghray, D. and Mikels-Carrasco, W.A. and Willaert, P. and Raybold, D.A. and Chawla, N.V.	USA	2014	A framework "eSeniorCare", which addresses all of these components together. It is a mobile platform that has the following features: medication scheduling, daily activity tracker. As a part of this framework, sessions are conducted to provide motivational lectures in aging.
15	Evaluating user perceptions of mobile medication management applications with older adults: A usability study	Grindrod, K.A. and Li, M. and Gates, A.	Canada	2014	MyMedRec, DrugHub, Pillboxie, and PocketPharmacist are apps for personal medication management.
16	Integrating Universal Design (UD) principles and mobile design guidelines to improve design of mobile health applications for older adults	Kascak, L.R. and Rebola, C.B. and Sanford, J.A.	USA	2014	Remote patient monitoring (RPM) is an app designed to record, visualize, and send data to the healthcare provider’s office. Providind remote monitor and communication between the chronic patient and healthcare provider is readily available.
17	MOBI-COG: A mobile application for instant screening of dementia using the mini-cog test	Nirjon, Shahriar and Emi, Ifat Afrin and Mondol, Md Abu Sayeed and Salekin, Asif and Stankovic, John A.	USA	2016	The MOBI-COG App is a complete automation of a widely used 3-minute dementia screening test called the Mini-Cog test, which is administered by primary caregivers for a quick screening of dementia in elderly.
18	Parkdetect: Early diagnosing parkinson's disease	Graca, Ricardo and Castro, Rui Sarmento E and Cevada, Joao	Portugal	2014	An app that allows tests to be performed to help diagnose Parkinson's Disease in the elderly.
19	SnapTag: Leveraging situated memory to enhance self-efficacy for well-being	Baadkar, Suraj and Singh, Gaurav and Saraf, Atul and Bagalkot, Naveen	India	2014	Snaptag is an app that leverages situated memory to enhance self-efficacy in senior citizens for their wellbeing.
20	Social networking-based personal home telehealth system: A pilot study	Huang, Y.-C. and Hsu, Y.-L.	Taiwan	2017	Care Deliver Frame (CDF) is an app that provide a personal home telehealth system based on social networking.
21	Smartphone based continuous monitoring system for home-bound elders and patients	Megalingam, Rajesh Kannan and Pocklassery, Goutham and Jayakrishnan, Vivek and Mourya, Galla and Thulasi, Athul Asokan	India	2015	An app that integrates a continuous monitoring system for the elderly in order to collect sensor data, identify critical conditions and alert the caregiver in case of emergency.
22	Quantification of postural stability in older adults using mobile technology	Ozinga, S.J. and Alberts, J.L.	USA	2014	Cleveland Clinic Balance Assessment App is designed to collect data from the user in order to quantity and quality postural stability in older adults.
23	Randomized controlled feasibility trial of two telemedicine medication reminder systems for older adults with heart failure	Goldstein, C.M. and Gathright, E.C. and Dolansky, M.A. and Gunstad, J. and Sterns, A. and Redle, J. D. and Josephson, R. and Hughes, J.W.	USA	2014	iRx Reminder is an app for medication adherence that provides medication reminders and acted as a passive medication-taking log for older adults.
24	Using a mobile health application to support self-management in COPD: a qualitative study	Williams, Veronika and Price, Jonathan and Hardinge, Maxine and Tarassenko, Lionel and Farmer, Andrew	United Kingdom	2015	An app to support self-management to improve quality of life in older adults with Chronic obstructive pulmonary disease.
25	A portable fall detection and alerting system based on k-NN algorithm and remote medicine	Jian, H. and Chen, H.	China	2017	An app that integrates a system of monitoring the elderly, that detect falls by capted data and send alert emergency.
26	Activity tracker and elderly: Usability and motivation of mobile healthcare in the context of elderly people	Rasche, Peter and Wille, Matthias and Theis, Sabine and Schafer, Katharina and Schlick, Christopher M. and Mertens, Alexander	Germany	2016	VitaDock + is an app that sychronizes informatin capted by a smartband in order to track activities.
27	Assist-Me, a volunteer mobile emergency system to assist elderly people	Mallat, Hady Khaddaj and Abdulrazak, Bessam	Canada	2014	Assist-me is a platform with two apps. The first one for elderly people that require emergency assistance and the second for caregivers volunteers who receive the request.
28	An efficient home-based risk of falling assessment test based on Smartphone and instrumented insole	Ayena, Johannes C. and Chapwouo T., Landry D. and Otis, Martin J-D. and Menelas, Bob-A.J.	Canada	2014	OLST is an app used for executate the clinical test One-Leg Standing. It records data from sensors to estimate user performance. The app allows a remote monitoring the ris of falls on the elderly.
29	An integrated caregiver-focused mHealth framework for elderly care	Ghazal, Mohammed and Khalil, Yasmina Al and Dehbozorgi, Fatemeh Jalil and Alhalabi, Marah Talal	United Arab Emirates	2018	An app that integrates an elderly care system, to provide a maintenance of health history, diet management, and detection of major fall accidents.
30	Design for personalized mobile health applications for enhanced older people participation	Devos, P. and Jou, A. Min and De Waele, G. and Petrovic, M.	Belgium	2018	An app able to record the medication intake, log physiological parameters and perform cognitive assessment.
31	Combining apps targeting professionals and senior citizens to improve housing accessibility and influence housing provision policies	Helle, T. and Iwarsson, S. and Lunn, T.B. and Iversen, M.H. and Jonsson, O. and Martensson, K. and Svarre, T. and Slaug, B.	Denmark	2014	This mobile app was developed for senior citizens to raise their awareness of possible accessibility problems in their current dwelling and in other apartments within the available housing stock.
32	Inferring loneliness levels in older adults from smartphones	Sanchez, Wendy and Martinez, Alicia and Campos, Wilfrido and Estrada, Hugo and Pelechano, Vicente	Mexico	2018	¡Vive! is an app capable to predict accuracies for social loneliness and existential crisis monitoring the older adult’s activities and determining his or her level of loneliness in relation of each factor.
33	Influence of mobile ICT on the adherence of elderly people with chronic diseases	Mertens, Alexander and Rasche, Peter and Theis, Sabine and Wille, Matthias and Schlick, Christopher and Becker, Stefan	Germany	2015	Medication Plan is an app which aims at supporting regular and correct intake of medication and documentation of vital parameters.
34	Health Promotion using Tablet Technology with Older Adult African American Methadone Clients: A Case Study	Brusoski, Melissa and Rosen, Daniel	USA	2017	Ovoo is an videoconference app that was used in this study to evaluate the feasibility of tablet for older adult in a educational initiative.
35	KeepSafe: Wristband device for heart-rate monitoring	Pinto, Vitor and Sousa, Raquel and Goncalves, Gil	Portugal	2017	A mobile application that integrates a system for heart rate monitoring by capted data through a smartband.
36	Detection of episodes of major depression in older adults through physiological markers and movement patterns: Case study	Edwing, Almeida C. and Marco, Ferruzca N. and Ivan, Guitierrez P.	Mexico	2016	A mobile application that integrates a system for heart rate monitoring through data captured using a wristband.
37	Evaluation of a mobile home care platform lessons learned and practical guidelines	Panagopoulos, Christos and Kalatha, Eirini and Tsanakas, Panayiotis and Maglogiannis, Ilias	Greece	2014	The study talnks about two applications, the first BioAssist is an app that integrates a health care system to communicate with the cloud-based platform services and also acts as a platform gateway to the various sensors and smartwatches and the other “iTriage” system enables users to look up symptoms and derive possible causes, treatment options procedures and common complications.
38	Handling of emergency situations with elderly patients using autonomous mobile robot and smart tablets	Mehdi, Syed Atif and Humayoun, Shah Rukh and Avtandilov, Artem and Berns, Karsten	Germany	2017	ESCoM app was developed as part of a system to facilitate the caregiver staff to collaborate each other and interact with the elderly person in an emergency situation alone at home for properly executing the rescue operation.
39	Design of a Mobile Social Community Platform for Older Chinese People in Urban Areas	Gao, Qin and Ebert, Daniel and Chen, Xing and Ding, Yao	China	2017	This work proposes an app called LonelyNo. The application facilitates the organization of leisure-time activities between older people with similar interests living in adjacent areas.
40	Multi-domain training in healthy old age: Hotel Plastisse as an iPad-based serious game to systematically compare multi-domain and single-domain training	Binder, J.C. and Zollig, J. and Eschen, A. and Merillat, S. and Rocke, C. and Schoch, S.F. and Jancke, L. and Martin, M.	Switzerland	2017	Hotel Plastisse is an iPad-based training tool for older adults that allows the comparison of the simultaneous training of spatial navigation, visuomotor function, and inhibition to the training of each of these functions separately.
41	Mobile Conversational Agents for Context-Aware Care Applications	Griol, D. and Callejas, Z.	Spain	2017	The authors proposed a framework that allows developing context-aware multimodal conversational agents. As a proof of concept, they developed an Android app for older adults suffering from Alzheimer’s. The app helps them to preserve their cognitive abilities and enhance their relationship with their environment.
42	Wearable sensor-based system to promote physical activity among elderly people	Sarria-Ereno, A. and Mendez-Zorrilla, A. and Garcia-Zapirain, B. and Gialelis, J.	Spain	2016	In this paper, it is proposed a system to promote physical activity among older people. As part of this system, the authors developed an app to receive the data sent by the sensors.
43	Smartphone-based gait measurement application for exercise and its effects on the lifestyle of senior citizens	Miura, Takahiro and Yabu, Ken-Ichiro and Hiyama, Atsushi and Inamura, Noriko and Hirose, Michitaka and Ifukube, Tohru	Japan	2015	The authors implemented an application to measure walking characteristics based on sensors embedded in a representative smartphone: the Apple iPhone 4S. The results indicate that the mobile application can help motivate seniors to walk more regularly and improve their walking ability
44	My Interventional Drug-Eluting Stent Educational App (MyIDEA): Patient-Centered Design Methodology	Boyd, Andrew Dallas and Moores, Kaitlin and Shah, Vicki and Sadhu, Eugene and Shroff, Adhir and Groo, Vicki and Dickens, Carolyn and Field, Jerry and Baumann, Matthew and Welland, Betty and Gutowski, Gerry and Flores, Jr., Jose D. and Zhao, Zhongsheng and Bahroos, Neil and Hynes, Denise M. and Wilkie, Diana J.	USA	2017	MyIDEA is an educational tool to supplement the education of the nurse and physician in medication adherence. MyIDEA was designed for patients with a sixth-grade reading level so that most patients could understand the information.
45	Security of personal bio data in mobile health applications for the elderly	Kim, Jong Tak and Kang, Un Gu and Lee, Young Ho and Lee, Byung Mun	Korea	2017	In this work, the authors investigated security in the transmission of health data. To validate the proposed secure transmission method, it was developed an mobile app with the following features: log-in, bio-information input, bio-information retrieval, query referral and configuration.
46	UPSA-M: Feasibility and initial validity of a mobile application of the UCSD Performance-Based Skills Assessment	Moore, R.C. and Fazeli, P.L. and Patterson, T.L. and Depp, C.A. and Moore, D.J. and Granholm, E. and Jeste, D.V. and Mausbach, B.T.	USA	2016	UPSA-M is an app to execute the test UCSD Performance-Based Skills Assessment (UPSA-M), a widely used test for assessing functional capacity in schizophrenia and other neurocognitively impaired patient populations.
47	Implementation of Android-based fall-detecting system	Jia, Huiyu and Li, Meihui and Ning, Yunkun and Liang, Shengyun and Li, Huiqi and Zhao, Guoru	China	2016	The authors proposed an application to detects falls promptly and minimize the damage to the aged. The system collect data from sensors in phone and then the data is analyzed by the fall detection algorithm.
48	Healthcare system for elders with automatic drug label detection	Tangtisanon, Pikulkaew	Thailand	2018	An Android app thats focuses on medicine taking system in order to give the elders better quality of life by solving major problems which are medicine taking and monitoring issue.
49	Design of Emotional Construction-Based Life Service APP for Elderly People	Qi, Xuan and Zhang, Wen and Hao, Fei	China	2017	Tongxin Bell's is an app design to reduce inconvenience caused by memory degradation in older adults. Your main function is to remind the older adults about their medicines, medical appointments, routine and so on. In other hand, the family of older adult can also benefit of the app to receive alerts about care activities for the older person.

Paper ID	Title	Authors	Country	Year	Description
50	Utility of a mHealth App for Self-Management and Education of Cardiac Diseases in Spanish Urban and Rural Areas	de Garibay, V.G. and Fernandez, M.A. and de la Torre-Diez, I. and Lopez-Coronado, M.	Spain	2018	This work analyzed the utility of a mobile health app named HeartKeeper in several groups of population. HeartKeeper has been developed to improve the patients' conditions by motivating them to do healthy activities and avoid bad habits.
51	Adoption and use of a mobile health application in older adults for cognitive stimulation	Yasini, M. and Marchand, G.	France	2016	The authors evaluated the adoption of the Stim'Art application that is dedicated to cognitive stimulation in the elderly. This app offers many serious games to work different cognitive functions like memory, attention or concentration.
52	6LoWPAN-enabled fall detection and health monitoring system with Android smartphone	Yi, Won-Jae and Sarkar, Oishee and Gonnot, Thomas and Monsef, Ehsan and Saniie, Jafar	USA	2014	The authors proposed an architecture of fall detection system. The fall detection algorithm performs real-time processing and analysis of the collected data. Additionally, the Wireless Intelligent Personal Communication Node is used to bridge the processed data analysis results to the Android smartphone through Bluetooth. Finally, the fall detection information is displayed in the app.
53	A longitudinal evaluation of the acceptability and impact of a diet diary app for older adults with age-related macular degeneration	Hakobyan, L. and Lumsden, J. and Shaw, R. and O'Sullivan, D.	United Kingdom	2016	A longitudinal field evaluation was conducted to investigate how the diet diary app it was received and adopted by older adults. The app seek to generate individualised dietary recommendations.
54	A mobile application improves therapy-adherence rates in elderly patients undergoing rehabilitation A crossover design study comparing documentation via iPad with paper-based control	Mertens, A. and Brandl, C. and Miron-Shatz, T. and Schlick, C. and Neumann, T. and Kribben, A. and Meister, S. and Diamantidis, C.J. and Albrecht, U.-V. and Horn, P. and Becker, S.	Germany	2015	This study investigated if the Medication Plan app affects the medication adherence in elderly patients. This app is able to set reminders for a number of medications, considering the drug intake needs of patients with chronic conditions on polypharmacy.
55	Acceptance of mobile technology by older adults: A preliminary study	Kim, S. and Gajos, K.Z. and Muller, M. and Grosz, B.J.	USA	2018	This work conducted a semi-structured interviews with 16 older adults who used a set of activity-tracking applications. With the results, the authors proposed an extension of the existing theoretical models that explains the technology acceptance behavior.
56	An mHealth Tool Suite for Mobility Assessment	Madhushri, Priyanka and Dzhagaryan, Armen and Jovanov, Emil and Milenkovic, Aleksandar	USA	2017	A suite of smartphone applications for assessing mobility in the elderly population. The suite includes smartphone applications that automate and quantify standardized medical tests for assessing mobility.
57	An analysis of application usage for notes and reminders by older persons-ElderNote Case study	De Melo, Josias E.A. and Rodrigues, Sandra Souza and Martins, Guilherme A. and Antonelli, Humberto Lidio and Fortes, Renata P.M. and Castro, Paula Costa	Brazil	2016	ElderNote is a reminder app designed for an older audience. This work used 21 brazilian older adults to evaluate the usage of this app. They concluded that it is accessible and have good usability.
58	Development of a Multi-Agent m-Health Application Based on Various Protocols for Chronic Disease Self-Management	Park, Hyun Sang and Cho, Hune and Kim, Hwa Sun	Korea	2017	SmPHR was developed aiming to ensure the interoperability of various personal health devices (PHDs) and electronic medical record systems (EMRs) for continuous selfmanagement of chronic disease patients. The SmPHR provides vital signs measurements, receiver information management, measured result outputs, and transmission functions for a chronic disease patient.
59	Development of a health care assistant App for the seniors	Tsai, W.-L. and Cheng, C.-F.	Taiwan	2018	The Health Care Assistant app can provide personal medication care, food care, and drug information for the patients. This app also is integrated with the national health systems of Taiwan. Thus, users can obtain the latest personal medication information.
60	Development of an experimental setup to investigate multimodal information representation and superposition for elderly users in healthcare context	Wille, Matthias and Seinsch, Tobias and Kummer, Rebecca and Rasche, Peter and Theis, Sabine and Brohl, Christina and Mertens, Alexander and Schlick, Christopher	Germany	2015	In this paper, it was proposed an experimental setup to test multimodal information representation and superposition for elderly users. To validate this setup, the authors developed a mobile application to perform different tests.
61	IoT Planting: Watering system using mobile application for the elderly	Lekjaroen, Krittin and Ponganantayotin, Rachatapon and Charoenrat, Arnon and Funilkul, Suree and Supasitthimethee, Umaporn and Triyason, Tuul	Thailand	2014	An Android application where the older adult can see a plant via monitoring menu which is a real-time communication between application and ip-camera.
62	HBPF: A Home Blood Pressure Framework with SLA guarantees to follow up hypertensive patients	Cuadrado, Josep and Vilaplana, Jordi and Mateo, Jordi and Solsona, Francesc and Solsona, Sara and Rius, Josep and Alves, Rui and Camafort, Miguel and Torres, Gerard and Betriu, Angels and Gutierrez, Josep M. and Fernandez, Elvira	Spain	2017	A cloud-based mobile app thats allows hypertensive patients to communicate with their health-care centers and facilitate following up hypertensive patient.
63	Design of a health care platform for the elderly	Cui, Jining and Song, Peipei and Li, Wenyu and Han, Shun and Li, Liang and Liu, Zhenqiang and Soo, Yew Guan and Zhu, Chi and Duan, Feng	China	2017	A mobile app thats receives a warning when a dangerous situation happens, and open the webcam to communicate with the user or remote control the platform.
64	Design of a mobile application to support non-pharmacological therapies for people with alzheimer disease	Reyes, Angie K. and Camargo, Jorge E. and Diaz, Gloria M.	Colombia	2015	A mobile application for helping caregivers in non-pharmacological therapies to early and moderate Alzheimer disease patients.
65	Fall detection using wearable accelerometers and smartphone	Basili, Luca and DeMaso-Gentile, Giuseppe and Scavongelli, Cristiano and Orcioni, Simone and Pirani, Stefano and Conti, Massimo	Italy	2014	An application crated to fall detection on the elderly and to send emergency alarms with the elder's localization.
66	Empowering the elderly: Implementation of navigation assistance application for public transportation	Heinonen, Samuli and Siira, Erkki	Finland	2017	The ASSISTANT is an app that provides Navigation assistance on the public transportation for the elderly. The app comunicates with the user via audio and visual guidance.
67	ESeniorCare: Technology for Promoting Well-Being of Older Adults in Independent Living Facilities	Dasgupta, Dipanwita and Reeves, Kimberly Green and Chaudhry, Beenish and Duarte, Mayra and Chawla, Nitesh V.	USA	2017	ESeniorCare is a tablet-based app that integrates various components of health and wellbeing for promoting to older adults an independent life.
68	Self-Monitoring of Health-Related Goals in Older Adults with Use of a Smartphone Application	Steinert, A. and Haesner, M. and Tetley, A. and Steinhagen-Thiessen, E.	Germany	2018	A app to enable self-Monitoring of Health-Related Goals for Older Adults
69	Parkinsons disease hand tremor detection system for mobile application	Fraiwan, L. and Khnouf, R. and Mashagbeh, A.R.	United Arab Emirates	2015	An app that measures the acceleration from the hand using a mobile cell phone accelerometer to detect and record rest tremor in Parkinson's disease patients.
70	Smartphone application for emergency signal detection	Figueiredo, Isabel N. and Leal, Carlos and Pinto, Luis and Bolito, Jason and Lemos, Andre	Portugal	2017	Knock-to-panic is an app for emergency signal detection that enables users to send an alarm signal to an emergency service, providing an unobtrusive method for elderly monitoring or safety protection.
71	Smartphone-based urine strip analysis	Anthimopoulos, Marios and Gupta, Sidharta and Arampatzis, Spyridon and Mougiakakou, Stavroula	Switzerland	2016	An app to automatically perform semi-quantitative colorimetric analysis on urine strips by using just one image of the strip, placed on a specially designed reference card designed for older adults and people with visual impairments.
72	Wireless Smart Health Monitoring System via Mobile Phone	Bakar, Nur Hidayah Binti Abu and Abdullah, Khaizuran and Islam, Md Rafiqul	Malaysia	2015	An app integrated to a health monitoring system that doctors and patients can use in order to monitor and check their condition via mobile phone.
73	Tablet-based support for older adults with severe mood disorders treated in an ambulatory geriatric psychiatry setting: Protocol of a feasibility study of the eCare@Home platform	Schuurmans, J. and van der Linden, J.L. and van Ballegooijen, W. and Ruwaard, J. and Stek, M.L. and Smit, J.H. and Riper, H.	Netherlands	2014	eCare@Home is a tablet-based self-management platform for late-life recurrent depression or bipolar disorder.
74	Older patients? use of technology for a post-discharge nutritional intervention ? A mixed-methods feasibility study	Lindhardt, T. and Nielsen, M.H.	Denmark	2015	A nutritional app witch combine goalsetting with self-monitoring and feedback. It enabled the patient to see photographs of the hospital menu including protein andenergy values for each course, to order directly from it and to recordhis/her intake.
75	SMAI - Mobile System for Elderly Monitoring	Stutzel, Matheus Costa and Fillipo, Michel and Sztajnberg, Alexandre and Brittes, Andre and Da Motta, Luciana Branco	Brazil	2016	An Android application thats monitor older adults patients with chronic degenerative disease that present functional loss.
76	Patient-centered tablet application for improving medication adherence after a drug-eluting stent	Shah, V. and Dileep, A. and Dickens, C. and Groo, V. and Welland, B. and Field, J. and Baumann, M. and Flores, J.D. and Shroff, A. and Zhao, Z. and Yao, Y. and Wilkie, D.J. and Boyd, A.D.	USA	2017	A patient-centered educational tablet application thats focused in increase in patient knowledge about dual antiplatelet therapy (DAPT) and medication possession ratio (MPR).
77	Design an interactive game app of horticultural therapy for older adults	Lai, Pin-Yi and Chen, Chien-Hsu	Taiwan	2015	A game app of horticultural therapy for aged people.
78	Helping the elderly with physical exercise: Development of persuasive mobile intervention sensitive to elderly cognitive decline	Alsaqer, Mohammed and Chatterjee, Samir	Saudi Arabia	2017	The App is called Adheree Booster (AdBo) and is designed to encourage, guide and monitor the progress of older people in their daily exercises.
79	Testing an app for reporting health concerns-Experiences from older people and home care nurses	Geransson, Carina and Eriksson, Irene and Ziegert, Kristina and Wengstrom, Yvonne and Langius-Eklof, Ann and Brovall, Maria and Kihlgren, Annica and Blomberg, Karin	Sweden	2014	The mobie application called Interaktor included direct access to self-care advice, graphs and a risk assessment model that sends alerts to nurses for rapid management.
80	A Conversational Medication Assistant for Heart Failure	Lobo, Joana and Ferreira, Liliana and Ferreira, Anibal J. S.	Portugal	2014	The personal medication advisor CARMIE in an android application developed as a conversational agent capable of interacting with users through spoken natural language, supporting users to manage information about their prescribed medicines.
81	A healthy lifestyle app for older adults with diabetes and hypertension: Usability assessment	Smith-Turchyn, J. and Gravesande, J. and Agarwal, G. and Mangin, D. and Javadi, D. and Peter, J. and Parascandalo, F. and Dolovich, L. and Richardson, J.	Canada	2017	The TAPESTRY-CM Healthy Lifestyle App is a healthy lifestyle app for older adults with diabetes and hypertension
82	A context-aware platform for comprehensive care of elderly people: Proposed architecture	Bravo-Torres, Jack F. and Ordonez-Ordonez, Jorge O. and Gallegos-Segovia, Pablo L. and Vintimilla-Tapia, Paul E. and Lopez-Nores, Martin and Blanco-Fernandez, Yolanda	Ecuador	2014	App to promote social interaction by recommending events preferred by the elder, which can be attended creating sporadic groups with people of similar tastes.
83	A new fall detection system on Android smartphone: Application to a SDN-based IoT system	Tran, Hai Anh and Ngo, Quynh Thu and Tong, Van	Vietnam	2016	App for fall detection based on a classification method to detect if it is a fall down or another common events (e.g. sitting, jumping, etc.).
84	A novel real-time fall detection system based on real-time video and mobile phones	Tong, Chao and Lian, Yu and Zhang, Yang and Xie, Zhongyu and Long, Xiang and Niu, Jianwei	China	2018	FallDetect is a system that combines an Android mobile application and a real-time video surveillance system to monitor elderly and detect fall events
85	A framework for evaluating mHealth tools for Older Patients on Usability	Wildenbos, G.A. and Peute, L.W. and Jaspers, M. W.M.	Netherlands	2016	App developed to support elderly in their hospital visit. The patients can view their appointments schedule and make use of a taxi transport service from home to the hospital and back.
86	A smartphone-based fall detection system for the elderly	Tsinganos, Panagiotis and Skodras, Athanassios	Greece	2017	App to fall detection focused on the elderly that can distinguish between falls and activities of daily living (ADL).
87	An assistive mobile application i-AIM app with accessible UI implementation for visually-impaired and aging users	Russ, Kan C. W.	Taiwan	2017	An app to support the mobility for the elderly that are visualy-impaired.
88	Android-based elderly support system	Saod, Aini Hafizah Mohd and Ghani, Siti Julia Amira Mat and Harron, Nur Athiqah and Ramlan, Siti Azura and Rashid, Anith Nuraini Abdul and Ishak, Nurul Huda	Malaysia	2017	An app that integrates a system for healthcare centers to monitor the elderly based on their location and detect the user's heart rate using a portable pulse sensor.
89	Design Jigsaw puzzle and app for Nostalgia-based support on elderly with Dementia	Chao, Fang-Lin and Feng, Chung-Shun and Fanjiang, Boxiu and Sun, Chang-Liang	Taiwan	2014	The app support the gameplay of a jigsaw puzzle nostalgie-based to the elderly with dementia.
90	An outdoor intelligent health care patient monitoring system	Veyilazhagan, R. and Bhanumathi, V.	India	2018	App to monitor patients suffering from chronic diseases, blood pressure and the elderly in their homes.
91	CONSIGNELA: A multidisciplinary patient-centered project to improve drug prescription comprehension and execution in elderly people and parkinsonian patient	Wanderley, Gregory Moro Puppi and Vandenbergh, Elodie and Abel, Marie-Helene and Barthes, Jean-Paul A. and Hainselin, Mathieu and Mouras, Harold and Lenglet, Aurelie and Tir, Melissa and Heurley, Laurent	France	2015	An app developed to better communicate medication prescriptions to older patients and patients with Parkinson's disease through the concept of virtual pillboxes.
92	Electronic health record for elderly patients	Michalik, Ilona and Kantoch, Anna	Poland	2017	An app created to assist health professionals in recording data of elderly patients from medical appointments. The application allows to execute, save, edit, view and send the results of medical appointments.
93	Informing caregivers through an assistive tool: An investigation of elderly care metrics	Klakegg, Simon and Van Berkel, Niels and Visuri, Aku and Luo, Chu and Goncalves, Jorge and Hosio, Simo and Huttunen, Hanna-Leena and Ferreira, Denzil	Finland	2016	An app aims to assist caregivers to provide a better service by raising awareness of the needs and daily routines of the elderly and to provide quick access to their well being.
94	Intervention Mapping Approach in the Design of an Interactive Mobile Health Application to Improve Self-care in Heart Failure	Athilingam, P. and Clochesy, J.M. and Labrador, M.A.	USA	2018	The app HeartMapp was designed to engage patients with heart failure in selfcare management by offering tailored alerts and feedback using mobile phones.

Paper ID	Title	Authors	Country	Year	Description
95	Evaluation of an app to support healthy living by older adults	Haslinda, Z. and Sani, A. and Petrie, H.	United Kingdom	2017	The app called MyHealthyLiving App was created to support older adults in maintaining good nutrition and hydration and to allow them to track their intake of fruit and vegetables and appropriate liquids.
96	Adoption and feasibility of a communication app to enhance social connectedness amongst frail institutionalized oldest old: an embedded case study	Neves, Barbara Barbosa and Franz, Rachel L. and Munteanu, Cosmin and Baecker, Ron	Australia	2015	An app to improve communication with and for older adults at risk of social isolation and loneliness.
97	Designing a context-aware assistive infrastructure for elderly care	Klakegg, Simon and Van Berkel, Niels and Visuri, Aku and Huttunen, Hanna-Leena and Hosio, Simo and Luo, Chu and Goncalves, Jorge and Ferreira, Denzil	Finland	2014	An app that integrates an IoT solution, CARE, which aims to provide daily support for elderly caregivers with a better understanding of elderly needs and ultimately, improve the care service.
98	Development and Evaluation of a Mobile Application Suite for Enhancing the Social Inclusion and Well-Being of Seniors	Goumopoulos, Christos and Papa, Ilia and Stavrianos, Andreas	Greece	2017	The system Senior App Suite integrates mobile computing combined with web and service-oriented technologies to offer a mobile application suite that seniors can easily use to access services, spanning various application areas such as social networking, emergency detection and overall well-being.
99	Communication technology adoption among older adult veterans: the interplay of social and cognitive factors	Leone, Cristina and Lim, Joan Soo Li and Stern, Anita and Charles, Jocelyn and Black, Sandra and Baecker, Ronald	Canada	2016	InTouch is an electronic communication platform designed to be accessible by computer naive seniors.
100	Elderly healthcare assistance application using mobile phone	Handojo, Andreas and Sutiono, Tioe Julio Adrian and Purbowo, Anita Nathania	Indonesia	2018	An app to help elderly people and their family member to supervise and monitor the health of the elderly. Its features are to monitor the location of the elderly,medication control, doctor appointment schedule, medical record records, emergency phone to family number or personal doctor, among others.
101	Human-Centered Design Study: Enhancing the Usability of a Mobile Phone App in an Integrated Falls Risk Detection System for Use by Older Adult Users	Harte, Richard and Quinlan, Leo R. and Glynn, Liam and Rodriguez-Molinero, Alejandro and Baker, Paul M. A. and Scharf, Thomas and OLaighin, Gearoid	Ireland	2016	Wiisel is a smartphone app to be used within a connected health fall risk detection system.
102	Life-support system for elderly as assistance in independent living	ele, Denis and Jurani, Nadja and Kouh, Ines and Debevc, Matja	Slovenia	2017	A project of mobile application which allows informal caregivers monitoring daily activities of elderly where data is received from remotesensors installed in elderly homes.
103	Managing heart failure on the Go: Usability issues with mHealth apps for older adults	Morey, Stephanie A. and Barg-Walkow, Laura H. and Rogers, Wendy A.	Australia	2018	Two apps called HeartPartner and Heart Failure Health Storylines created to facilitate the management of Congestive Heart Failure (CHF) by older adults.
104	Medication management apps: Usable by older adults?	Stuck, Rachel E. and Chong, Amy W. and Mitzner, Tracy L. and Rogers, Wendy A.	USA	2018	Medisafe is a medication management app. In this study, the authors evaluated if this app was accessible to older adults.
105	MHealth based ubiquitous fall detection for elderly people	Bhati, Neha	India	2015	Fall detection is an app that integrates a embedded system to monitor older adults and detect falls in indoor and outdoor environments. The system has the capability of generating alarm and informing the healthcare provider and relatives or friends in emergency situations.
106	Mobile application for elderly assistance in public transport	Concepcion-Sanchez, Jose a. and Suarez-Armas, Jonay and Caballero-Gil, Pino and alvarez-Diaz, Nestor	Spain	2016	Transport Guide App is an app that integrates an operation of a embedded system to guide older adults through the means of transport in a simpler way, calculating the optimal route for the journeys that users want to do.
107	Mobile application on healthy diet for elderly based on persuasive design	Salim, M.H.M. and Ali, N.M. and Noah, S.A.M.	Malaysia	2018	Nutrihealth is an application with information on Body Mass Index (BMI), that suggest menus, and calories intake to provide a behaviour change and a healthy diet for older adults.
108	Usability testing of the iPhone app to improve pain assessment for older adults with cognitive impairment (Prehospital Setting): A qualitative study	Docking, R.E. and Lane, M. and Schofield, P.A.	Australia	2014	iPhone pain assessment is an app created to support clinical paramedic practice to improve pain assessment of older adults with cognitive impairment.
109	The Health Buddies App as a Novel Tool to Improve Adherence and Knowledge in Atrial Fibrillation Patients: A Pilot Study	Desteghe, Lien and Kluts, Kiki and Vijgen, Johan and Koopman, Pieter and Dilling-Boer, Dagmara and Schurmans, Joris and Dendale, Paul and Heidbuchel, Hein	Belgium	2018	Health Buddies is an application created inform and to handle an incentive strategy to improve the adherence of elderly people with atrial fibrillation to their care routine.
110	Mobile Phone Intervention for Heart Failure in a Minority Urban County Hospital Population: Usability and Patient Perspectives	Dang, S. and Karanam, C. and Gomez-Orozco, C. and Gomez-Marin, O.	USA	2017	An app to disease management program for patients with Chronic heart failure using short message service (SMS).
111	SousChef: Mobile meal recommender system for older adults	Ribeiro, David and Machado, Joao and Ribeiro, Jorge and Vasconcelos, Maria Joao M. and Vieira, Elsa F. and De Barros, Ana Correia	Portugal	2014	SousChef is an application capable of creating a personalized meal plan based on the information provided by the user. The nutritional recommendations and the application have been thought and designed for older adults.
112	Design and Evaluation of a Medication Adherence Application with Communication for Seniors in Independent Living Communities	Dasgupta, D. and Johnson, R.A. and Chaudhry, B. and Reeves, K.G. and Willaert, P. and Chawla, N. V.	USA	2016	A tablet-based application designed specifically for seniors to track their medications and a web portal for their care providers to track medication adherence.
113	New Hick's law based reaction test App reveals information processing speed better identifies high falls risk older people than simple reaction time	Qiu, Hai and Xiong, Shuping	South Korea	2015	An app to execute a reaction test, assessing cognitive function related fall risks in older people.
114	Monitoring of the daily living activities in smart home care	Vanus, Jan and Belesova, Jana and Martinek, Radek and Nedoma, Jan and Fajkus, Marcel and Bilik, Petr and Zidek, Jan	Czech Republic	2015	A mobile application that communicates with a monitoring environment of daily living activities (ADL).
115	Personal Health Assistance for Elderly People via Smartwatch Based Motion Analysis	Lutze, Rainer and Waldhor, Klemens	Germany	2017	An app to smartwatches witch analyzes the motion patterns of the older adult to provide personal health assistance with he features of communication, orientation, localization and health hazard detection.
116	PlaIMoS: A Remote Mobile Healthcare Platform to Monitor Cardiovascular and Respiratory Variables	Miramontes, Ramses and Aquino, Raul and Flores, Arturo and Rodriguez, Guillermo and Anguiano, Rafael and Rios, Arturo and Edwards, Arthur	Mexico	2018	An app that integrates an embedded solution to monitor cardiovascular and respiratory variables.
117	The rehabilitation enhancing aging through connected health (REACH) study: study protocol for a quasi-experimental clinical trial	Ni, Meng and Brown, Lorna G. and Lawler, Danielle and Ellis, Terry D. and Deangelis, Tamara and Latham, Nancy K. and Perloff, Jennifer and Atlas, Steve J. and Percac-Lima, Sanja and Bean, Jonathan F.	USA	2016	A tablet-based app to deliver a progressive home-based exercise program emphasizing lower-extremity function and a walking program.
118	User-centered evaluations with older adults: Testing the usability of a mobile health system for heart failure self-management	Cornet, V.P. and Daley, C.N. and Srinivas, P. and Holden, R.J.	USA	2018	Engage is an app witch supports the setting and logging of self-management goals, recording and tracking of self-management data such as vitals and symptoms, and provides learning tips about heart failure self-management.
119	Ticket to talk: Supporting conversation between young people and people with dementia through digital media	Welsh, Daniel and Morrissey, Kellie and Foley, Sarah and McNaney, Roisin and Salis, Christos and McCarthy, John and Vines, John	United Kingdom	2016	Ticket to Talk is a mobile application designed to stimulate talk between young people and older people with dementia.
120	Mobile device based smart medication reminder for older people with disabilities	Mohammed, H.B.M. and Ibrahim, D. and Cavus, N.	Cyprus	2017	NEU-MED is an app witch his main functionality is to remind the user to take correct medication at the correct times of the day.
121	A Mobile App (iBeni) With a Neuropsychological Basis for Cognitive Stimulation for Elderly Adults: Pilot and Validation Study	Martinez-Alcala, Claudia I. and Rosales-Lagarde, Alejandra and Hernandez-Alonso, Esmeralda and Melchor-Agustin, Roberto and Rodriguez-Torres, Erika E. and Itza-Ortiz, Benjamin A.	Mexico	2017	iBeni is a mobile app for cognitive stimulation implemented among a group of elderly adults. The app aims to improve cognitive functions and to decelerate the impairment process in healthy older adults or older adults with mild indicators of decline.
122	A Mobile Health Intervention to Reduce Pain and Improve Health (MORPH) in Older Adults With Obesity: Protocol for the MORPH Trial	Fanning, Jason and Brooks, Amber K. and Ip, Edward and Nicklas, Barbara J. and Rejeski, W. Jack	USA	2015	In this work, the authors proposed the MORPH: a mobile health intervention to reduce pain and improve health in older adults with obesity
123	Activity Monitors as Support for Older Persons' Physical Activity in Daily Life: Qualitative Study of the Users' Experiences	Ehn, Maria and Eriksson, Lennie Carlen and Akerberg, Nina and Johansson, Ann-Christin	Sweden	2018	The aim of this study was to investigate how seniors experience using activity monitors (AMs) as support for PA in daily life. Two commercially available bracelets for monitoring PA were used in the tests, namely Withings Activité Pop (Withings) and Jawbone UP3 (Jawbone). The authors concluded that activity monitors can be valuable for supporting seniors' physical activity. However, the potential of the solutions for a broader group of seniors can significantly be increased.
124	Design of Wisdom Home System for the Aged Living Alone	Ma, Ying and Li, Jianxing and Liu, Lisang	China	2017	This paper presents a system to control home environments for the older living alone. The system can realize the local, WEB and mobile terminal APP intelligent home control.
125	Context-Aware, Accurate, and Real Time Fall Detection System for Elderly People	Muheidat, Fadi and Tawalbeh, Lo'Ai and Tyrer, Harry	USA	2018	In this research, it is presented a Context-aware and private real-time fall detection system (FDS) for elderly people. The proposed system consists of sensor pad placed under a carpet; the electronics reads walking activity to provide an automated health monitoring and alert system. The users can obtain these results and make decisions by accessing the cloud through his/her mobile devices and in real time manner.
126	Context-aware mobile app for the multidimensional assessment of the elderly	Berrocal, Javier and Garcia-Alonso, Jose and Murillo, Juan M. and Mendes, David and Fonseca, Cesar and Lopes, Manuel	Spain	2017	This work proposes an app that gathers the elderly contextual information, stores it and, periodically, assesses the elder's functional status, sending the result to the health team. And a health application, which receives the assessment results.
127	Development of a path to home mobile app for the geriatric rehabilitation program at bruyère continuing care: Protocol for user-centered design and feasibility testing studies	Backman, C. and Harley, A. and Peyton, L. and Kuziemsky, C. and Mercer, J. and Monahan, M.A. and Schmidt, S. and Singh, H. and Gravelle, D.	Canada	2015	The Path to Home mobile app was used to manage the personalized needs of geriatric rehabilitation patients during their transitions from hospital to home.
128	Development of elderly reminder mobile application using mental model	Baharum, Aslina and Ismail, Rozita and Saad, Nordin and Daruis, Dian Darina Indah and Noh, Nor Azida Mohamed and Noor, Noorsidi Aizuddin Mat	Malaysia	2016	The Elderly Reminder mobile application was developed using the user-centred design (UCD) approach. Different of many others reminders, the proposed app focus on user interface for elderly users.
129	Efficacy of bingocize?: A game-centered mobile application to improve physical and cognitive performance in older adults	Shake, M.C. and Crandall, K.J. and Mathews, R.P. and Falls, D.G. and Dispennette, A.K.	USA	2016	This work evaluated the Bingocize, a game-centered mobile app that can combine bingo with healthy activities such as exercise and/or health topic education.
130	Engaging older adults to inform diabetes medication adherence mobile application selection	Conway, Cheryl M. and Kelechi, Teresa J. and Nemeth, Lynne S.	USA	2018	In this work, two apps were evaluated considering their usage for diabetes medication adherence: OnTimeRx and Medisafe. The OnTimeRx is a mobile reminder designed by a pharmacist that delivers reminders by Short Message Service (SMS), email, or phone. The Medisafe operates on an iOS or Android mobile system by providing medication reminders.
131	A field study of older adults with cognitive impairment using tablets for communication at home: Closing technology adoption gaps using InTouch	Yurkewich, A. and Stern, A. and Alam, R. and Baecker, R.	Canada	2016	InTouch is a mobile application that consists of a wrapper library to modify the Google Gmail interface,and has been adapted through field study evidence to improve its usability specifically for older adults. It was evaluated in this research through a case study with older adults with cognitive impairment.
132	Feasibility of virtual tablet-based group exercise among older adults in Siberia: Findings from two pilot trials	Nikitina, S. and Didino, D. and Baez, M. and Casati, F.	Russian Federation	2018	The objective of this paper was to study the feasibility of home-based online group training. The authors used a trainee app provided by Gymcentral. The main features are: training program, online group exercising, persuasion strategies, remote monitoring and feedback, and communication feature.
133	Adapting a Psychosocial Intervention for Smartphone Delivery to Middle-Aged and Older Adults with Serious Mental Illness	Whiteman, K.L. and Lohman, M.C. and Gill, L.E. and Bruce, M.L. and Bartels, S.J.	USA	2016	The authors proposed a smartphone application for integrated medical and psychiatric selfmanagement intervention .
134	Enabling aid in remote care for elderly people via mobile devices: The MobiCare case study	Garcia, Anderson C. and De Lara, Silvana M. A.	Brazil	2018	Mobicare app helps to schedule medications, remind about favorite TV shows and send audio messages. In this work, the authors evaluated the acceptance of Mobicare in the care of digitally disengaged elderly people
135	Does culture affect usability? A trans-European usability and user experience assessment of a falls-risk connected health system following a user-centred design methodology carried out in a single European country	Stara, V. and Harte, R. and De Rosa, M. and Glynn, L. and Casey, M. and Hayes, P. and Rossi, L. and Mirelman, A. and Baker, P.M.A. and Quinlan, L.R. and OLaighin, G.	Italy	2017	The purpose of WIISEL was to continuously assess fall risk by measuring gait and balance parameters associated with fall risk, and to detect falls. In this paper, it was reported the results of a usability and user experience (UX) assessment of the WIISEL system in multiple countries.
136	Healthcare and security system for elderly and disabled people using ARM Microcontroller	Birajdar, Abhishek Kashinath and More, Prajakta	India	2018	This work proposes an app that gathers the elderly data (like location, temperature and pulse rate) from SMS sent by the microcontroller. In case of emergency, the app can show the exact location of patient.

Paper ID	Title	Authors	Country	Year	Description
137	Long-term care: How to improve the quality of life with mobile and e-health services	Delmastro, Franca and Dolciotti, Cristina and Palumbo, Filippo and Magrini, Massimo and Di Martino, Flavio and La Rosa, Davide and Barcaro, Umberto	Italy	2018	INTESA is a suite of technological tools (like mobile apps) and services that assist older people to offer a more independent, healthy, comfortable, safe, and socially engaged life.
138	Mini home-based vital sign monitor with android mobile application (myVitalGear)	Yusof, Mas Azalya and Hau, Yuan Wen	Malaysia	2018	This paper proposes a home-based vital sign monitor, named myVitalGear. The myVitalGear has a mobile application to display the vital sign measurement, and sending of notification message and user location to the healthcare provider if any abnormality is detected.
139	Mobile assisted living: Smartwatch-based fall risk assessment for elderly people	Haescher, Marian and Matthies, Denys J.C. and Srinivasan, Karthik and Bieber, Gerald	Germany	2017	This work presents a smartwatch application that allows the computation of multiple fall risk related parameters. A major focus group for this approach are elderly people.
140	Personalizing health-related ICT interface and application: Older adults and elderly caregivers preferences	Dos Santos, Mariane M.T. and Antonelli, Humberto Lidio and Rodrigues, Sandra Souza and De Silva, Caroline L.O. and Fortes, Renata P. M. and Castro, Paula Costa	Brazil	2016	The authors developed the app called ANNI to study the interest, preferences and problems with a skin mobile application for personalizing it with health older adults and social networking of dependent elderly individuals
141	Translating Behavior Change Principles Into a Blended Exercise Intervention for Older Adults: Design Study	Mehra, Sumit and Visser, Bart and Dadema, Tessa and van den Helder, Jantine and Engelbert, Raoul H. H. and Weijs, Peter J. M. and Krose, Ben J. A.	Netherlands	2016	MBvO (More Exercise for Seniors) app was used in this work to identify theory-based components of a blended intervention that supports older adults to exercise at home.
142	The ActiveAgeing Mobile App for Diabetes Self-management: First Adherence Data and Analysis of Patients? in-App Notes	Triberti, Stefano and Bigi, Sarah and Rossi, Maria Grazia and Caretto, Amelia and Laurenzi, Andrea and Dozio, Nicoletta and Scavini, Marina and Pergolizzi, Enrico and Ozzello, Alessandro and Serino, Silvia and Riva, Giuseppe	Italy	2017	ActiveAgeing is a mobile app supporting daily self-management. The app was developed as a tool to be integrated within the therapeutic alliance between doctors and patients and aiming at increasing patients' awareness of their condition and their decision-making autonomy.
143	Mobile follow-up system for elderly and disabled people	Vera, Pablo Martin and Carrau, Mariano Kaimakamian and Rodriguez, Rocio Andrea	Argentina	2016	This paper presents a mobile app prototype for planned care and follow-up of elderly or disabled people. The app uses the sensors in devices such as GPSs, accelerometers, barometers, etc, and enables a noninvasive control and follow-up system.
144	SousChef: Improved meal recommender system for Portuguese older adults	Ribeiro, D. and Ribeiro, J. and Vasconcelos, M.J. M. and Vieira, E.F. and de Barros, A.C.	Portugal	2017	SousChef app is a meal recommender system. The nutritional recommendations and the application was thought and designed for older adults, presenting friendly user interfaces and following the guidelines of a nutritionist.
145	Sensor fusion for recognition of activities of daily living	Wu, J. and Feng, Y. and Sun, P.	USA	2018	This work proposes an Activity of daily living (ADL) Recognition System. As part of this system, they present the ADL Recorder App that runs on a user's smartphone with multiple embedded sensors.
146	The Development and Acceptability of a Mobile Application for Tracking Symptoms of Heart Failure among Older Adults	Portz, J.D. and Vehovec, A. and Dolansky, M.A. and Levin, J.B. and Bull, S. and Boxer, R.	USA	2014	The authors developed an application called HF app to allow patients to track their symptoms of Heart Failure. The HF app was designed to allow older adults with HF to record their weight, log their symptoms, and symptom severity.
147	Improving pain treatment with a smartphone app: Study protocol for a randomized controlled trial	Suso-Ribera, C. and Mesas, A. and Medel, J. and Server, A. and Marquez, E. and Castilla, D. and Zaragoza, I. and Garcia-Palacios, A.	Spain	2016	In this work the Pain Monitor app was used for telemonitoring adults with chronic pain. The goal of this study was to explore the effects of using a smartphone apps to monitor this health condition.
148	RehabPartner: Motion tracking assistant using a novel complementary feedback filter	Lim, S.L. and Yean, S. and Lee, B.S. and Kiat, Y.C.	Singapore	2016	RehabPartner is an application that is designed to assist the elderly in rehabilitation exercises. The authors proposed a Complementary Feedback Filter (CFF) to fuse readings from the smartphone's noisy motion sensors in order to get accurate orientation of body segments. The CFF was combined with the RehabPartner app.
149	Self-Management of Chronic Diseases Among Older Korean Adults: An mHealth Training, Protocol, and Feasibility Study	Kim, Heejung and Park, Eunhee and Lee, Sangeun and Kim, Mijung and Park, Eun Jeong and Hong, Soyun	Korea	2017	This work used many personal health tracking apps (Cardiio, iCare, Hypertension Protector, Diabetes Guide, Diabetes Note, Noom Coach) to analyze the feasibility of delivering mHealth training protocols to elderly individuals.
